# Impact of liver cirrhosis etiology on results of diagnostic tests for minimal hepatic encephalopathy

**DOI:** 10.1038/s41598-026-49607-8

**Published:** 2026-04-22

**Authors:** Julius Felix Martin Egge, Alena Friederike Ehrenbauer, Maria Magdalena Gabriel, Anja Tiede, Jana Al-Ayoubi, Jennifer Witt, Jim Benjamin Mauz, Martin Andreas Kabelitz, Lea Sophie Wagner, Heiner Wedemeyer, Anika Grosshennig, Gerrit Maximilian Grosse, Benjamin Maasoumy, Karin Weissenborn

**Affiliations:** 1 Department of Neurology, Hannover Medical School, Carl-Neuberg Str. 1, 30625 Hannover, Germany; 2 Department of Gastroenterology, Hepatology, Infectious Diseases and Endocrinology, Hannover Medical School, Carl-Neuberg Str. 1, 30625 Hannover, Germany; 3 German Center for Infection Research (DZIF), Hannover/Braunschweig, Germany; 4 Hannover Medical School, Institute of Biostatistics, Carl-Neuberg Str. 1, 30625 Hannover, Germany; 5 Department of Neurology, University Hospital Basel, Petersgraben 4, Basel, 4031 Switzerland

**Keywords:** PSE syndrome test, Animal naming test, Critical flicker frequency, Cognitive impairment, Etiology, Complications of liver cirrhosis, Diseases, Gastroenterology, Medical research

## Abstract

The influence of cirrhosis etiology on minimal hepatic encephalopathy (mHE) testing and prediction of overt hepatic encephalopathy (oHE) remains unclear. We investigated the impact of cirrhosis etiology on mHE testing and its predictive value for oHE. In this observational study, 312 patients with liver cirrhosis underwent six mHE tests: Portosystemic Encephalopathy Test yielding the PHES, Animal Naming Test, Critical Flicker Frequency, Inhibitory Control Test, EncephalApp, and Continuous Reaction Time. Etiologies included alcohol-related liver disease (ALD), metabolic dysfunction-associated steatohepatitis (MASH), metabolic/alcohol-associated liver disease (MetALD), infectious/autoimmune liver disease (IALD), and cryptogenic/other (CRYO). Multivariable regression models adjusted for relevant confounders were used to analyze etiology-related differences. Predictive value for oHE was assessed over one year using competing risk models. Patients with IALD performed significantly better on PHES than ALD patients (adjusted β: 1.8, [0.5-3]). PHES scores were comparable across steatotic etiologies. PHES showed moderate predictive value for oHE in steatotic (ALD sHR: 2.2 [1.0–4.9]; MASH: 3.4 [1.3–8.8]; MetALD: 2.5 [0.8–8.0]) but strong predictive value in non-steatotic etiologies (IALD sHR: 5.7 [2.2–14.7]; CRYO: 5.8 [1.2–29.3]) Other tests showed only minor etiology-related differences. Cirrhosis etiology may influence PHES results and its predictive value for oHE.

**Supplementary Information:**

The online version contains supplementary material available at 10.1038/s41598-026-49607-8.

## Introduction

 Cognitive impairment (CogIm) is a major reason for poor health-related quality of life, reduced social activity and low socio-economic potential of patients with liver cirrhosis^1,2^. Unfortunately, CogIm is common among these patients^3–5^. The causes are manifold^6–8^. In most cases, minimal hepatic encephalopathy (mHE) is suspected, although many patients with liver cirrhosis show other causes of CogIm too, such as psychiatric, neurological or internal comorbidities. Differentiating the genesis of CogIm is difficult or even impossible^8–10^. The question to which extent the etiology of liver cirrhosis impacts cognition has been a matter of debate since the discovery of minimal or subclinical hepatic encephalopathy (HE)^11^. To mention, all neuropsychological tests applied for diagnosing mHE are not specific for mHE but aim to detect CogIm which is - in patients with liver cirrhosis - often linked to HE^9^.

It is undisputed that long-term alcohol abuse, regardless of the presence of liver disease, can negatively affect brain function. The mechanisms are manifold and can be exacerbated by liver cirrhosis, such as thiamine deficiency resulting in Wernicke’s encephalopathy, for example^12^. In recent decades, the impact of other etiologies of liver cirrhosis upon brain function has been increasingly discussed. Felipo et al. showed that pre-cirrhotic metabolic dysfunction-associated steatohepatitis (MASH) patients achieved worse results on Portosystemic Encephalopathy (PSE) Syndrome Test than healthy controls^13^. Furthermore, patients with metabolic dysfunction associated steatotic liver disease (MASLD) present microglia activation, and neuroinflammation^14,15^ and patients with underlying cholestatic disease or Hepatitis C virus (HCV) infection exhibit cognitive impairments - even in non-cirrhotic states of liver disease^16,17^.

Usually, patients with liver cirrhosis are tested for the presence of brain dysfunction by application of neurocognitive tests^5,18^. These tests are also used for predicting the occurrence of an overt hepatic encephalopathy (≥ HE grade 2, oHE) episode. However, the influence of liver cirrhosis etiology on these test results is rarely studied^3,19^ and when it is, patients are only considered dichotomously: with or without alcohol-related liver disease (ALD)^20–22^.

The aim of this study was to assess the impact of liver cirrhosis etiology upon the results of six commonly used mHE tests. Additionally, its potential effect on the value of mHE tests for predicting oHE within one year follow-up was investigated.

## Results

### Classification of the etiologies of liver cirrhosis

To examine the influence of liver cirrhosis etiology on mHE test results, patients were separated into five groups. Steatotic liver disease (SLD) was separated into ALD, MASH and metabolic and alcohol related liver disease (MetALD). Due to the rarity of autoimmune hepatitis, cholestasis associated liver disease or HCV as etiology of liver cirrhosis, we decided to bundle these patients in one group, as these patients in non-cirrhotic stages of liver disease have comparable deficits in neurocognitive tests as well as similar microstructural alterations of the brain according to quantitative magnetic resonance imaging (qMRI)^16,17^. In the following we summarize this group under infectious or autoimmune liver disease (IALD). In Suppl. Table 1, we have listed all etiologies combined in this group and their respective frequencies. Lastly, we summarized patients with cryptogenic or other etiologies of cirrhosis than those aforementioned as CRYO.

### Baseline characteristics

The majority of patients was male (70%); median MELD 12 (IQR: 9–16) and median Child-Pugh Score 7 (IQR: 6–9). A history of oHE was present in 92 patients (30%). Median years of school education were 10 (IQR: 9–11) and median age 59 years (IQR: 51–65). The predominant etiology was ALD (111/312, 36%). Seventy of these 111 patients (63%) had been abstinent for at least 6 months prior to testing. Fifty-two patients had MASH (17%), 41 MetALD (13%), and 63 patients IALD (20%) as etiology of cirrhosis. Forty-two (14%) patients had CRYO. A liver biopsy was performed in 43% of the patients. Forty-four patients had a transjugular intrahepatic portosystemic shunt (TIPS) insertion at baseline (14%, median time with TIPS before testing: 7 months (IQR: 2–16)). These patients were compared with those without TIPS in Suppl. Table 2.

Table 1 shows the baseline characteristics of the study cohort.

**Table 1:** Baseline characteristics.

Number of patients	All patients	ALD	MASH	MetALD	IALD	CRYO	Missing %	*p*-value
	312	111	52	41	63	45		
Age	59 [51, 65]	57 [51.5, 63.5]	61 [54.75, 67]	62 [53, 69]	55 [44, 61]	54 [45, 65]		**0.001**
Sex male	217 (70%)	79 (71%)	33 (64%)	35 (85%)	37 (59%)	33 (73%)		0.177
Child-Pugh-Score	7 [6, 9]	8 [6, 9]	6 [5, 8]	8 [6, 9]	7 [6, 8.75]	6 [5, 8]	2.2	**0.025**
MELD	12 [9, 16]	12 [9, 14]	11 [9, 16]	13 [10, 17]	12 [9, 17]	10 [8, 14]	0.3	0.330
TIPS at baseline	44 (14%)	21 (19%)	8 (15%)	3 (7%)	10 (16%)	2 (4%)		0.117
Inpatients	241 (77%)	91 (82%)	37 (71%)	36 (88%)	49 (78%)	28 (62%)		**0.028**
Years of school education	10 [9, 11]	10 [9, 11]	10 [9, 10]	10 [10, 10]	10 [9, 11]	10 [9, 11]		0.28
Diabetes mellitus	93 (30%)	21 (19%)	33 (64%)	16 (39%)	14 (22%)	9 (20%)		**< 0.001**
Diabetes controlled	68 (73%)	16 (76%)	22 (67%)	12 (75%)	10 (71%)	8 (89%)		0.738
Cardiovascular disease	126 (40%)	36 (32%)	33 (64%)	28 (68%)	15 (24%)	14 (31%)		**< 0.001**
German as native language	259 (83%)	98 (88%)	45 (87%)	40 (98%)	53 (84%)	23 (51%)		**< 0.001**
Any HE prophylaxis	194 (62%)	87 (78%)	28 (54%)	23 (56%)	36 (57%)	20 (44%)		**< 0.001**
Lactulose intake	165 (53%)	73 (66%)	25 (48%)	19 (46%)	32 (51%)	16 (36%)		**0.007**
Rifaximin intake	88 (28%)	38 (34%)	15 (29%)	13 (32%)	13 (21%)	9 (20%)		0.237
LOLA intake	44 (14%)	17 (15%)	8 (15%)	9 (22%)	6 (10%)	4 (9%)		0.356
Change in HE prophylaxis during FU	117 (38%)	47 (42%)	15 (29%)	21 (51%)	21 (33%)	13 (29%)		0.093
Any HE prophylaxis EoF	223 (72%)	92 (83%)	36 (69%)	34 (83%)	38 (60%)	23 (51%)		**< 0.001**
Lactulose intake EoF	207 (66%)	86 (78%)	33 (64%)	31 (76%)	35 (56%)	22 (49%)		**0.002**
Rifaximin intake EoF	131 (42%)	61 (55%)	17 (33%)	18 (44%)	22 (35%)	13 (29%)		**0.007**
LOLA intake EoF	52 (17%)	16 (14%)	12 (23%)	10 (24%)	8 (13%)	6 (13%)		0.311
Previous oHE episode	92 (30%)	37 (33%)	13 (25%)	12 (29%)	18 (29%)	12 (27%)		0.733
Liver biopsy	133 (43%)	20 (18%)	26 (50%)	12 (29%)	52 (83%)	23 (51%)		**< 0.001**
Lab values								
Sodium (mmol/l)	137 [134, 139]	136 [133, 139]	137.5 [135, 139]	135 [134, 138]	137 [135, 139.5]	137 [134, 140]		0.221
Creatinine (µmol/l)	90 [74, 117.25]	90 [76.5, 126]	91.5 [70.75, 126.25]	110 [84, 150]	84 [67, 108.5]	84 [76, 97]		**0.002**
CHE (kU/l)	3.05 [2, 4.37]	2.64 [1.97, 4.42]	3.52 [2.36, 4.85]	3 [1.94, 3.64]	2.9 [1.94, 3.84]	3.35 [2.56, 4.98]	3.5	0.095
Bilirubin (µmol/l)	21 [13, 45]	20 [12.5, 37.5]	20.5 [15, 36]	18 [10, 37]	31 [15.5, 71.5]	20 [13, 45]		**0.041**
Albumin (g/dl)	33 [28, 38]	32 [27, 38]	34 [28, 39]	35 [30, 38]	32 [28, 35]	34 [28.5, 39]	2.2	0.192
White blood cells (tsd/µl)	5.1 [3.6, 7]	6.1 [4.35, 7.8]	3.95 [3, 6.05]	5.4 [4.3, 6.9]	5.1 [3.25, 7.25]	4.2 [3.4, 5.6]		**< 0.001**
Platelets (tsd/µl)	101 [62.5, 155.5]	127 [86.5, 193.5]	80.5 [57.75, 108.5]	111 [73, 146]	87 [56, 144.75]	63 [53, 130]	0.3	**0.001**
Hemoglobin (g/dl)	10.7 [9.28, 12.9]	10.5 [8.75, 12.85]	10.8 [9.47, 12.6]	9.7 [8.6, 12.2]	10.9 [9.5, 12.6]	12.6 [10.5, 13.6]		**0.018**
INR	1.24 [1.11, 1.37]	1.21 [1.08, 1.33]	1.24 [1.15, 1.46]	1.22 [1.12, 1.35]	1.26 [1.12, 1.37]	1.25 [1.12, 1.42]	0.3	0.418

All continuous variables are presented as median and interquartile range, dichotomous values are presented as absolute and relative frequencies, Kruskal-Wallis test for continuous variables, χ-2 for dichotomous values, values of *p* < 0.05 are highlighted in bold font.

Abbreviations: ALD: alcohol-related liver disease, CHE: cholinesterase, CRYO: cryptogenic/other, EoF: end of follow-up HE: hepatic encephalopathy, IALD: infectious/autoimmune liver disease, INR: international normalized ratio, MASH: metabolic dysfunction-associated steatohepatitis, MELD: model for end-stage liver disease, MetALD metabolic and alcohol-associated liver disease, LOLA: L-ornithine-L-aspartate, oHE: overt hepatic encephalopathy, TIPS: transjugular intrahepatic portosystemic shunt.

### Comparison of mHE test results in the five patient groups with different cirrhosis etiologies

Psychometric Hepatic Encephalopathy Score (PHES) test results in the 5 groups of etiology, were as follows: ALD (median − 4), MetALD (median: -4), MASH (median: -4), CRYO (median: -4), and IALD (median: -2, *p* = 0.012). A dichotomous view on the PHES test results showed that patients with IALD had the lowest frequency of abnormal test results (18/63, 29%), while patients with ALD and MetALD had the highest frequency (ALD: 54/111, 49%; MetALD 20/41, 49%; *p* = 0.048, Table 2).

**Table 2:** Test results at baseline.

	All patients	ALD	MASH	MetALD	IALD	CRYO	*p*-value
PHES	− 4 [− 7, − 1.75]	− 4 [− 8, − 2]	− 4 [− 8, − 1]	− 4 [− 8, − 2]	− 2 [− 5, − 1]	− 4 [− 6, − 2]	0.012
PHES abnormal (<− 4)	128 (41%)	54 (49%)	22 (42%)	20 (49%)	18 (29%)	14 (31%)	**0.048**
CRT Index	1.91 [1.46, 2.32]	2.10 [1.57, 2.43]	1.77 [1.50, 2.29]	1.78 [1.30, 2.52]	1.85 [1.48, 2.28]	1.77 [1.40, 2.23]	0.615
CRT abnormal	113 (49%)	31 (38%)	19 (54%)	13 (54%)	25 (52%)	25 (60%)	0.170
Stroop Off+OnTime (sec)	185.53 [161.87, 208.28]	187.14 [161.91, 205.12]	187.68 [179.72, 212.05]	207.83 [182.97, 224.13]	172.32 [146.98, 198.96]	190.06 [165.35, 212.21]	**0.011**
Stroop OffTime (sec)	85.67 [76.28, 96.43]	83.11 [76.12, 93.17]	87.69 [82.55, 101.36]	96.00 [79.00, 105.54]	78.69 [67.98, 88.31]	87.74 [79.59, 98.58]	**0.001**
Stroop OnTime (sec)	100.47 [85.22, 113.14]	100.43 [85.38, 112.87]	101.33 [93.92, 115.25]	110.39 [98.18, 119.05]	93.02 [77.99, 109.08]	99.68 [85.56, 111.80]	**0.048**
Stroop abnormal	111 (50%)	39 (51%)	17 (47%)	15 (65%)	16 (34%)	24 (60%)	0.071
ANT (animals/minute)	22 [19, 27]	24 [20, 29]	22 [19, 26.5]	22 [17.75, 25.5]	23 [19.75, 27]	20 [16, 25.75]	0.13
ANT z-Score	− 0.23 [− 1.06, 0.6]	0 [− 0.7, 0.68]	− 0.16 [− 0.58, 0.42]	− 0.43 [− 0.97, 0.54]	− 0.24 [− 0.92, 0.57]	− 0.56 [− 1.58, 0.5]	0.254
ANT abnormal	45 (20%)	12 (15%)	5 (14%)	3 (13%)	9 (19%)	16 (38%)	**0.02**
ICT weighted lures	17.86 [9.89, 30.26]	18.52 [10.2, 30.76]	23.4 [10.49, 33.97]	15.39 [11.63, 26.44]	15.73 [6.63, 27.53]	17.69 [11.82, 38.9]	0.504
ICT abnormal	67 (31%)	22 (30%)	12 (34%)	6 (26%)	11 (25%)	16 (39%)	0.656
CFF (in Hz)	41.8 [38.5, 46.75]	41.5 [38, 45.82]	42.4 [39.05, 48.04]	40.93 [37.53, 44.28]	41.5 [38.4, 46.9]	42.98 [39.28, 47.55]	0.382
CFF abnormal	60 (22%)	23 (24%)	8 (18%)	9 (26%)	14 (25%)	6 (15%)	0.737

All continuous variables are presented as median and interquartile range, dichotomous values are presented as absolute and relative frequencies, Kruskal-Wallis test for continuous variables, χ2 for dichotomous values, values of *p* < 0.05 are highlighted in bold font.

Abbreviations: ALD: alcohol-related liver disease, ANT: Animal Naming Test, CFF: Critical Flicker Frequency, CRT: Continuous Reaction Time Test, CRYO: cryptogenic/other, IALD: infectious/autoimmune liver disease, ICT: Inhibitory Control Test, MASH: metabolic dysfunction-associated steatohepatitis, MetALD metabolic and alcohol-associated liver disease, PHES: Portosystemic Hepatic Encephalopathy Score.

**Fig. 1:**
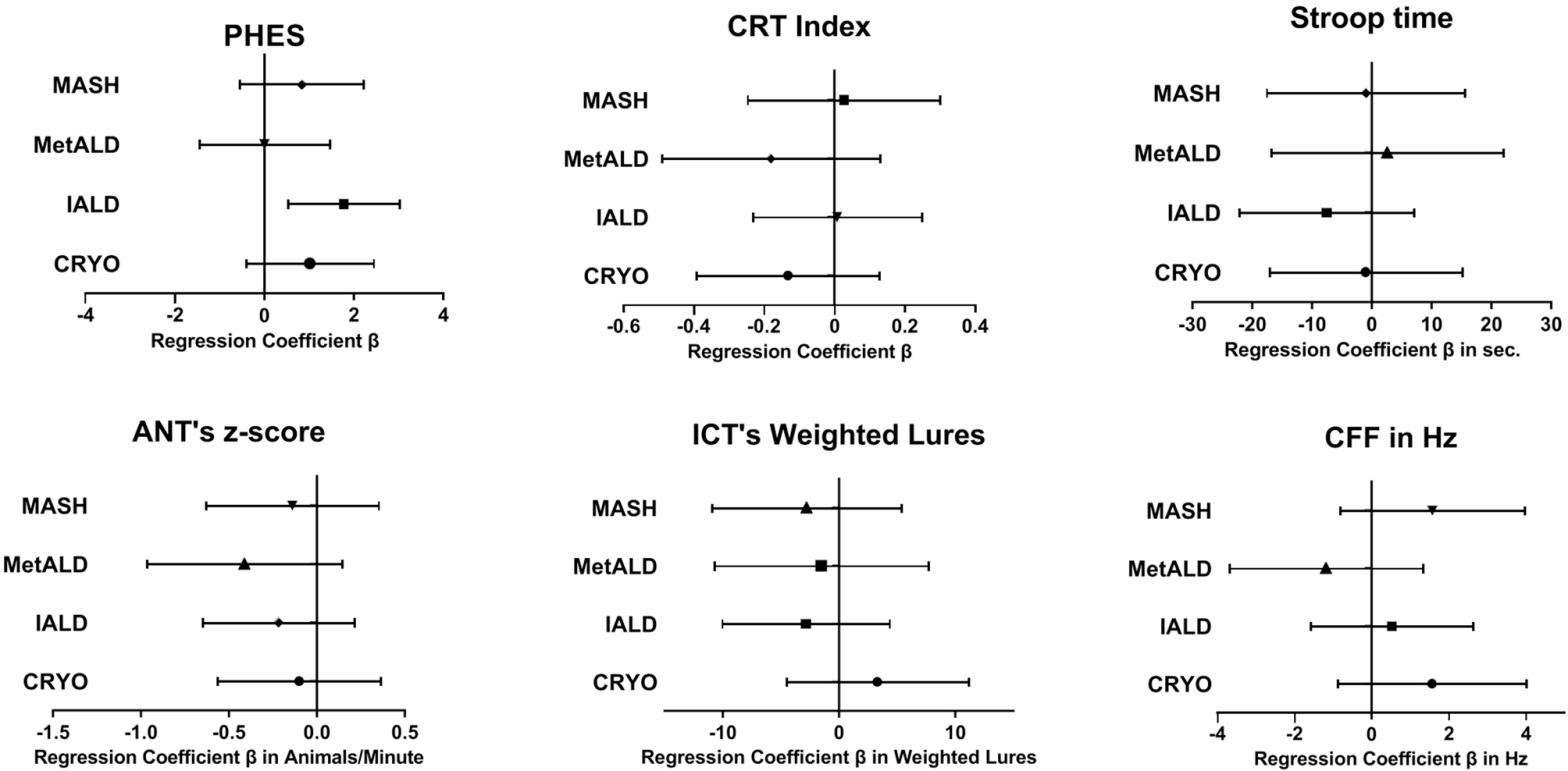
Multivariable linear regression analyses for mHE test results, Model A. Multivariable linear regression model with ALD as reference group, adjusted for sex, age, native language, and presence of diabetes mellitus. Pictured are point estimates and 95% confidence intervals for adjusted regression coefficient β. Abbreviations: ANT: Animal Naming Test, CFF: Critical Flicker Frequency, CRT: Continuous Reaction Time Test, ICT: Inhibitory Control Test, CRYO: cryptogenic & others, IALD: infectious or autoimmune liver disease, MASH: metabolic dysfunction-associated steatohepatitis, MetALD: metabolic dysfunction and alcohol associated steatotic liver disease, mHE: minimal hepatic encephalopathy, Hz: Hertz, PHES: Portosystemic Hepatic Encephalopathy Score.

**Fig. 2:**
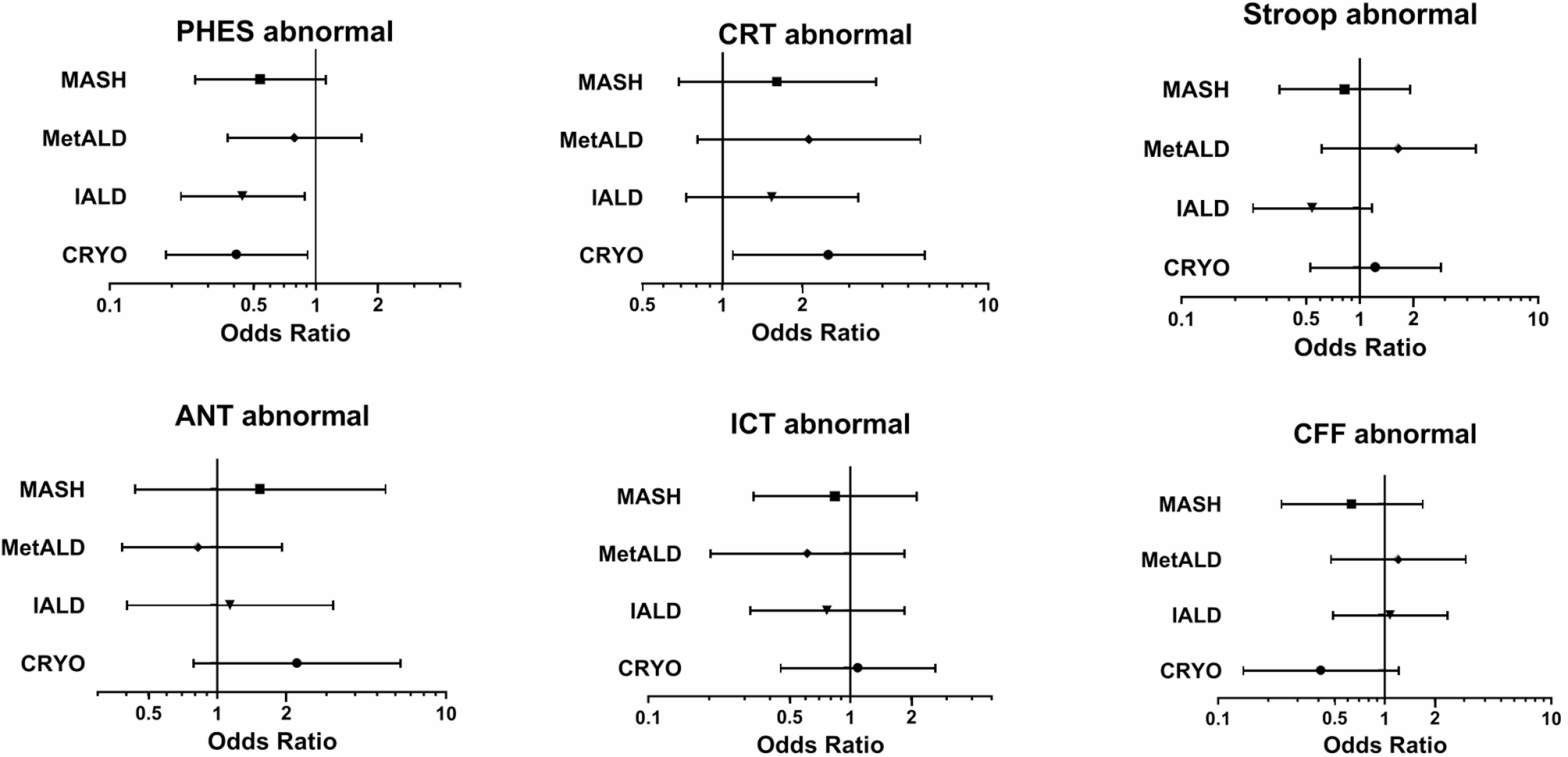
Multivariable binary logistic regression analyses for abnormal mHE test results, Model A. Multivariable binary logistic regression model adjusted for sex, age, native language, and presence of diabetes mellitus. ALD as reference group. Pictured are point estimates and 95% confidence intervals for adjusted Odds Ratio. Abbreviations: ANT: Animal Naming Test, CFF: Critical Flicker Frequency, CRT: Continuous Reaction Time Test, CRYO: cryptogenic & others, IALD: infectious or autoimmune liver disease, ICT: Inhibitory Control Test MASH: metabolic dysfunction-associated steatohepatitis, MetALD: metabolic dysfunction and alcohol associated steatotic liver disease, mHE: minimal hepatic encephalopathy, PHES: Portosystemic Hepatic Encephalopathy Score.

In linear regression analyses, patients with IALD had a regression coefficient β (RCβ) of 1.9 (95% CI [0.7–3.2], *p* = 0.002) in PHES compared to patients with ALD. Patients with IALD thus scored 1.9 points better in PHES than patients with ALD. All other etiologies performed comparable to ALD patients (Suppl. Table 3a). In multivariable analyses, adjusting for presence of diabetes mellitus, age, sex, and native language (Model A), patients with IALD showed an adjusted RCβ (aRCβ) of 1.8 [0.5-3] in comparison to ALD patients (*p* = 0.005). The same is evident in Model B, which included all variables that showed statistically significant group differences in the univariable analyses (see Methods). IALD patients showed an aRCβ of 1.8 ([0.6–3] *p* = 0.003, Suppl. Table 3b). All other etiologies performed comparably to ALD (Table 3, Suppl. Table 3a & b, Fig. 1). A similar result can be noted in binary logistic regression analyses. In the multivariable approach of Model A, patients with IALD showed an adjusted odds ratio (aOR) of 0.4 [0.2–0.9] for having an abnormal PHES result (*p* = 0.019). In this model, CRYO patients also show a significantly reduced aOR of 0.4 [0.2–0.9] (*p* = 0.028, Table 4, Suppl. Table 4a, Fig. 2). In Model B, only IALD patients remain with a significantly lower probability of an abnormal PHES (aOR 0.5 [0.2–0.9], *p* = 0.029, Suppl. Table 4b).

**Table 3:** Multivariable linear regression analyses for mHE test results at baseline, Model A.

	PHES		CRT index		Stroop off + on time		ANT’s z-score		ICT‘s weighted lures		CFF in Hz	
	aRCβ (95% CI)	*p*-value	aRCβ (95% CI)	*p*-value	aRCβ (95% CI)	*p*-value	aRCβ (95% CI)	*p*-value	aRCβ (95% CI)	*p*-value	aRCβ (95% CI)	*p*-value
MASH	0.846 (− 0.538 to 2.23)	0.23	0.027 (− 0.247 to 0.3)	0.849	− 1.02 (− 17.599 to 15.559)	0.904	− 0.14 (− 0.631 to 0.351)	0.575	− 2.792 (− 10.937 to 5.353)	0.5	1.578 (− 0.819 to 3.976)	0.196
MetALD	0.002 (− 1.457 to 1.461)	0.998	− 0.181 (− 0.491 to 0.129)	0.252	2.587 (− 16.84 to 22.015)	0.793	− 0.412 (− 0.967 to 0.143)	0.145	− 1.527 (− 10.745 to 7.691)	0.744	− 1.182 (− 3.686 to to 1.321)	0.353
IALD	**1.769 (0.524–3.014)**	**0.005**	0.008 (− 0.232 to 0.248)	0.947	− 7.582 (− 22.146 to 6.982)	0.306	− 0.218 (− 0.648 to 0.212)	0.318	− 2.862 (− 10.064 to 4.339)	0.434	0.523 (− 1.586 to 2.632)	0.626
CRYO	1.017 (− 0.41 to 2.443)	0.162	− 0.132 (− 0.392 to 0.127)	0.316	− 1.034 (− 17.153 to 15.085)	0.899	− 0.101 (− 0.566 to 0.364)	0.668	3.293 (− 4.547 to 11.134)	0.408	1.568 (− 0.879 to 4.015)	0.208

Multivariable linear regression model with ALD as reference group, adjusted for sex, age, native language, and presence of diabetes mellitus. All variables are shown as adjusted regression coefficient ß with 95% confidence interval in parentheses, p-values < 0.05 are highlighted in bold font.

Abbreviations: ANT: Animal Naming Test, aRCβ: adjusted regression coefficient β, CFF: Critical Flicker Frequency, CRT: Continuous Reaction Time Test, CRYO: cryptogenic/other, IALD: infectious/autoimmune liver disease, ICT: Inhibitory Control Test, MASH: metabolic dysfunction-associated steatohepatitis, MetALD metabolic and alcohol-associated liver disease, PHES: Portosystemic Hepatic Encephalopathy Score.

**Table 4:** Multivariable binary logistic regression analyses for abnormal mHE test results at baseline, Model A.

	PHES abnormal		CRT abnormal		Stroop abnormal		ANT’s z-Score abnormal		ICT abnormal		CFF abnormal	
	aOR (95% CI)	*p*-value	aOR (95% CI)	*p*-value	aOR (95% CI)	*p*-value	aOR (95% CI)	*p*-value	aOR (95% CI)	*p*-value	aOR (95% CI)	*p*-value
MASH	0.537 (0.259–1.113)	0.094	1.605 (0.683–3.774)	0.278	0.822 (0.352–1.915)	0.649	1.537 (0.436–5.418)	0.503	0.836 (0.33–2.116)	0.705	0.634 (0.239–1.683)	0.36
MetALD	0.784 (0.371–1.66)	0.526	2.109 (0.803–5.536)	0.13	1.64 (0.607–4.433)	0.33	1.666 (0.382–7.265)	0.497	0.611 (0.203–1.84)	0.381	1.202 (0.474–3.047)	0.698
IALD	**0.44 (0.221–0.876)**	**0.019**	1.535 (0.73–3.228)	0.258	0.541 (0.251–1.166)	0.117	1.135 (0.402–3.207)	0.811	0.766 (0.318–1.846)	0.552	1.075 (0.486–2.379)	0.858
CRYO	**0.411 (0.186–0.907)**	**0.028**	**2.503 (1.092–5.741)**	**0.03**	1.219 (0.526–2.826)	0.644	2.23 (0.783–6.308)	0.133	1.088 (0.45–2.631)	0.852	0.413 (0.141–1.213)	0.108

Multivariable binary logistic regression model adjusted for sex, age, native language, and presence of diabetes mellitus. All variables are shown as adjusted Odds Ratio with 95% confidence interval in parentheses, p-values < 0.05 are highlighted in bold font.

Abbreviations: ANT: Animal Naming Test, aOR: adjusted Odds Ratio, CFF: Critical Flicker Frequency, CRT: Continuous Reaction Time Test, CRYO: cryptogenic/other, IALD: infectious/autoimmune liver disease, ICT: Inhibitory Control Test, MASH: metabolic dysfunction-associated steatohepatitis, MetALD metabolic and alcohol-associated liver disease, PHES: Portosystemic Hepatic Encephalopathy Score.

Interestingly, abnormal Continuous Reaction Time Test (CRT) test results were less frequent in ALD patients (26/81, 38%) than in the other groups (most frequent CRYO 25/42, 60%, *p* = 0.17, Table 2). Multivariable binary logistic regression yielded a significant aOR for an abnormal CRT in patients with CRYO compared to patients with ALD in both Model A (aOR 2.5 [1.1–5.7], *p* = 0.03, Table 4, Fig. 2) and Model B (aOR 3.2 [1.4–7.4], Suppl. Table 4b). All other etiologies performed comparably to ALD, both in the binary logistic regression and in the linear regression (Tables 3 & 4, Figs. 1 and 2, Suppl. Table 3a & b, Suppl Table 4a & b).

When considering the percentage of abnormal test results in EncephalApp, providing the Stroop test, MetALD patients performed worst while IALD patients performed best (Table 2). In univariable analyses, patients with IALD showed an RCβ of -15.2 [-31-0.6] in comparison to patients with ALD (*p* = 0.073, Suppl. Table 3a) and remained insignificant after adjustment in multivariable analyses (Model A: aRCβ: -7.6 [-22.2-7] *p* = 0.306, Model B: -6.9 [-21.2-7.5] *p* = 0.346, Table 3, Suppl. Table 3b, Fig. 1). In binary logistic regression, all etiologies performed comparably to ALD patients (Table 4, Suppl. Table 4a & b, Fig. 2).

In Animal-Naming Test (ANT), all groups named a similar number of animals (worst: CRYO, median 20; best: ALD, median 24). Patients with CRYO had most frequently abnormal test results (16/42, 38%) compared to all other etiologies (13–19%, *p* = 0.02, Table 2). It should be noted here that a high number of CRYO patients were non-native speakers (21/42, 50%, Table 1). A poorer ANT z-score was strongly associated with native language other than German. In multivariable linear and binary logistic regression analyses, all etiologies performed comparably to patients with ALD (Tables 3, 4, Suppl. Table 3a & b Suppl. Table 4a & b, Figs. 1 and 2).

Inhibitory Control Test’s (ICT) Weighted Lures were associated with higher age and lower years of school education. MASH patients had numerically the worst result (Table 2). In multivariable analyses, all etiologies performed comparable to patients with ALD (Tables 3, 4, Suppl. Table 3a & b Suppl. Table 4a & b, Figs. 1 and 2).

In Critical Flicker Frequency (CFF), MetALD patients had the highest rate of abnormal test results (9/35, 26%, Table 2). In univariable linear regression and binary logistic regression, higher Child-Pugh Score was associated with abnormal CFF. Again, uni- and multivariable regression analyses revealed no impact of the underlying etiology (Tables 3, 4, Suppl. Table 3a,b Suppl. Table 4a,b, Figs. 1 and 2).

Suppl. Figure 1 shows the test results as boxplots grouped according to etiology for a concise presentation of the test results.

### Predictive value of PHES regarding the underlying etiology

**Fig. 3:**
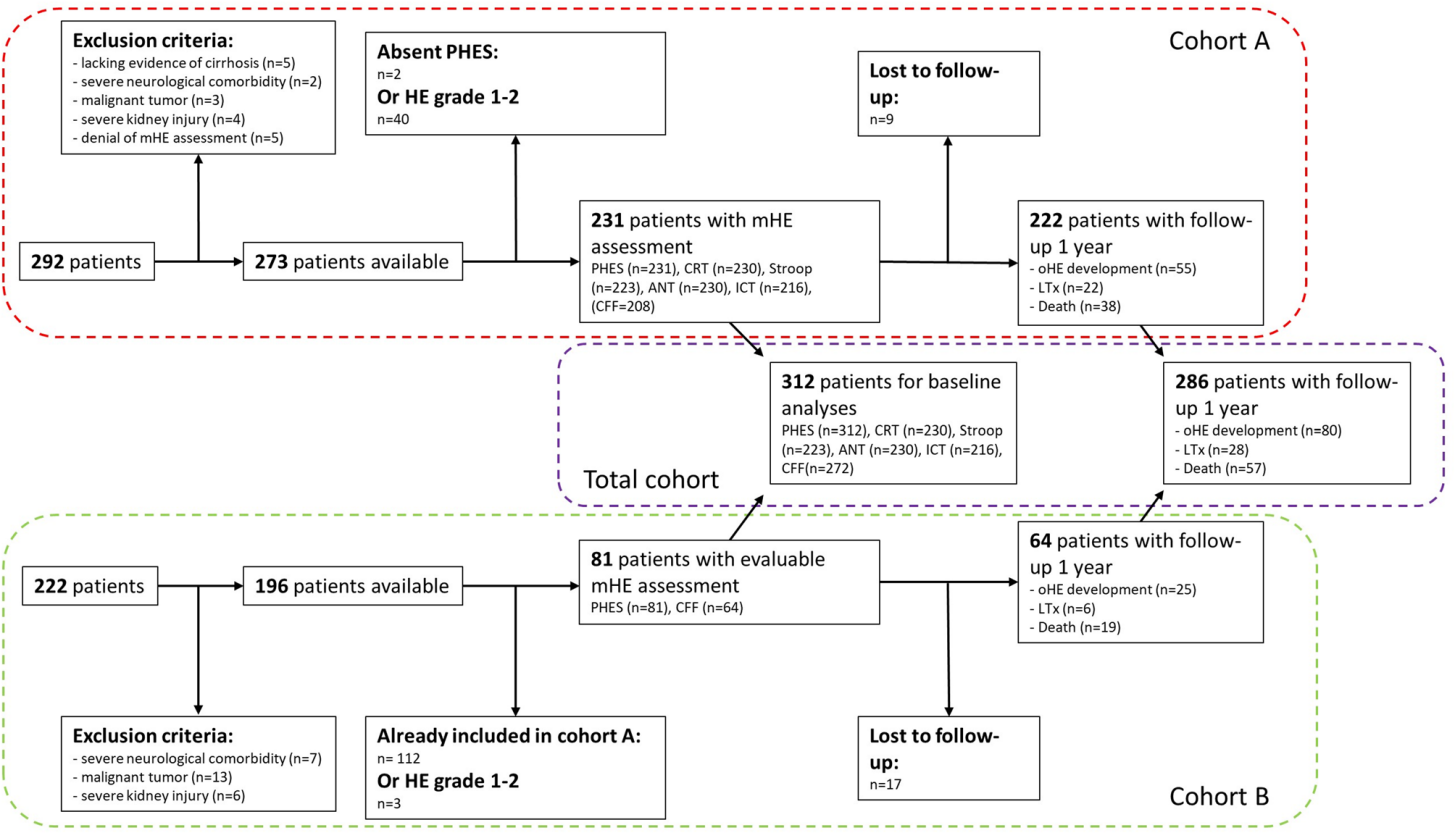
Study design. Abbreviations: ANT: Animal Naming Test, CFF: Critical Flicker Frequency, CRT: Continuous Reaction Time Test, HE: hepatic encephalopathy, ICT: Inhibitory Control Test LTx: liver transplantation, mHE: minimal hepatic encephalopathy, oHE: overt hepatic encephalopathy, PHES: Portosystemic Hepatic Encephalopathy Score.

During one-year of follow-up, 27 ALD (24%), 17 MASH (33%), 11 MetALD (26%), 18 IALD (29%), and 7 CRYO (16%) patients experienced an oHE episode (Suppl. Table 5). Additionally, 57 died (18%) and 28 underwent liver transplantation (LTx, 9%). Unfortunately, 26 patients were lost to follow-up (8%, Fig. 3).

**Fig. 4:**
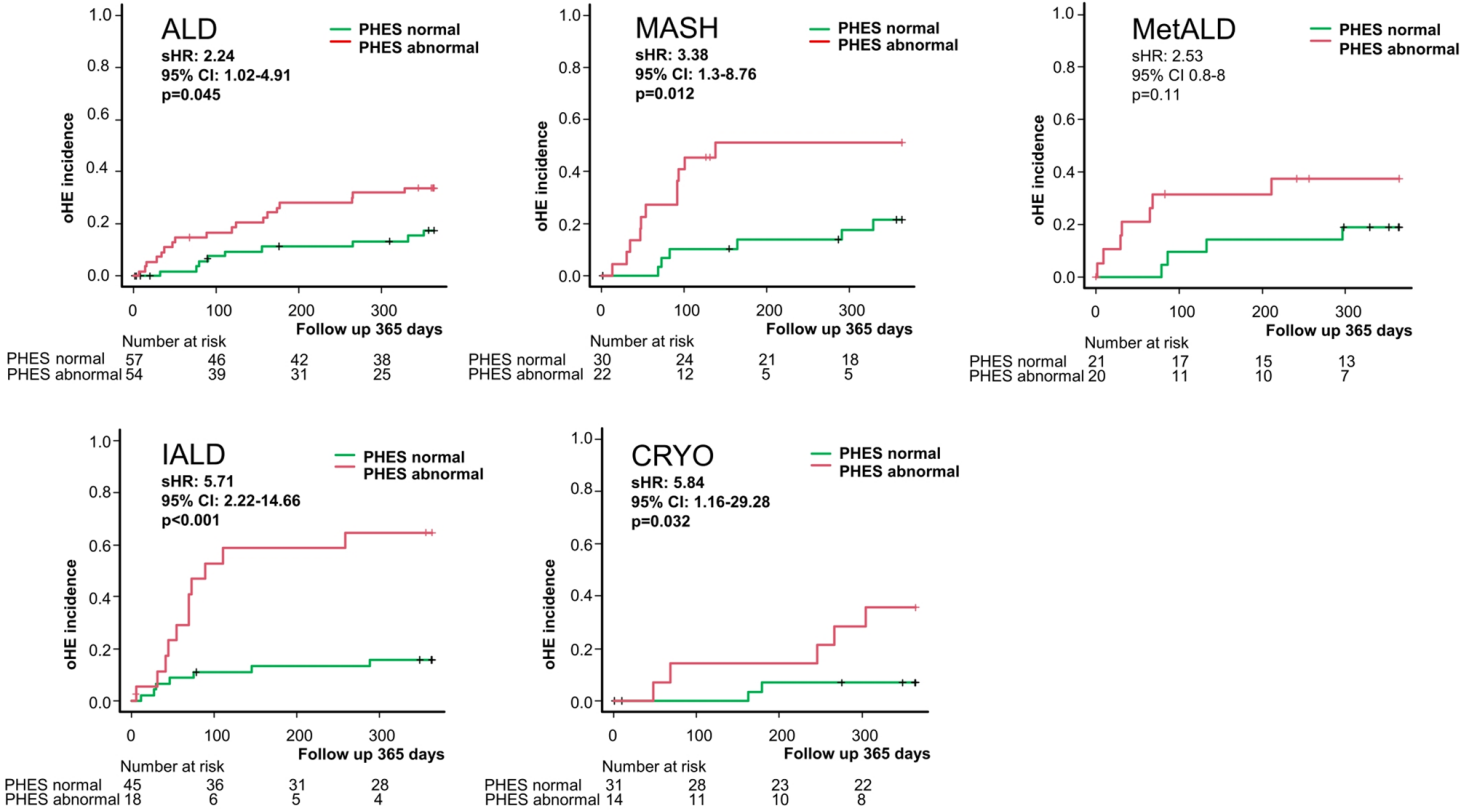
Fine and Gray’s competing risk analyses for the predictive value of an abnormal PHES for overt HE during a 365-day follow-up period with regard to the underlying etiology. Fine and Gray’s Competing Risk analyses for development of oHE during 365 days of follow-up. Death and liver transplantation as competing events. Abbreviations: ALD: alcohol-associated liver disease, CI: confidence interval, CRYO: cryptogenic & others, IALD: infectious or autoimmune liver disease, MASH: metabolic dysfunction-associated steatohepatitis, MetALD: metabolic dysfunction and alcohol associated steatotic liver disease, oHE: overt hepatic encephalopathy, PHES: portosystemic hepatic encephalopathy score, sHR: subdistribution hazard ratio.

To investigate the extent to which the etiology of cirrhosis influences the predictive value of PHES for oHE during a one-year follow-up period, we performed Fine & Gray competing risk analyses to examine the extent to which the subdistribution hazard ratio (sHR) for developing oHE with abnormal PHES differs depending on etiology. Patients with SLD showed the lowest predictive value of abnormal PHES for oHE during the 365 days of follow-up (ALD: sHR 2.2 [1-4.9] *p* = 0.045, MetALD: sHR 2.5 [0.8-8] *p* = 0.11, MASH: sHR 3.4 [1.3–8.8] *p* = 0.012). In contrast, patients with IALD or CRYO demonstrate a significantly higher predictive value of an abnormal PHES (IALD: sHR 5.7 [2.2–14.7] *p* < 0.001, CRYO: sHR 5.8 [1.2–29.3] *p* = 0.032, Fig. 4).

Additionally, we wanted to examine whether the optimal cut-off of the PHES for predicting oHE varies between the different etiologies. For this purpose, we performed maximally selected rank statistics (maxstat). In patients with ALD the best suitable estimated cut-off of the PHES to predict oHE is -8 (*p* = 0.02). In patients with MASH and MetALD, the optimal, albeit insignificant, cut-off is -5 (*p* = 0.08 and *p* = 0.57, respectively). In IALD patients, a cut-off of -4, was appropriate (*p* < 0.001) whereas for CRYO patients a cut-off of -6 (*p* = 0.029) seems suitable (Suppl. Figure 2).

## Discussion

Cognitive impairment in patients with liver cirrhosis is frequent, especially in patients with SLD^3,15^. In this study, we demonstrate that the results of diagnostic tests for mHE in patients with liver cirrhosis are influenced by the underlying etiology. Specifically, patients with IALD performed significantly better in the widely used reference standard for diagnosing mHE (PSE-Syndrome test) compared to those with ALD. This suggests that etiology may play a major role in the cognitive outcome of liver cirrhosis patients^23^.

In addition, our results indicate that mHE tests, particularly the PHES, are more relevant for predicting oHE in non-SLD. In steatotic conditions such as ALD, MASH and MetALD, the predictive capacity of the PSE-Syndrome test appears to be less effective.

The poorer performance of ALD patients compared to IALD patients in the PSE-Syndrome test could originate from the toxic effect of long-term alcohol abuse on cognitive function in addition to the effects of portal hypertension and liver cirrhosis^23^. This is hardly surprising from a pathophysiological point of view, as alcohol toxicity affects similar regions of the brain as HE. This applies in particular to the cerebellum, where early damage from HE occurs on the one hand, but where long-term alcohol abuse also leaves its mark^24,25^. Both can result in dysmetria, tremor and further cerebellar symptoms^25,26^. The comparable performance of ALD, MetALD and MASH patients fits in well with the current zeitgeist of research on metabolic syndrome and CogIm. Here, too, a serious influence on cognition has been observed in recent years, primarily due to neuroinflammation and reduced synaptic density in the brain, resulting in impaired memory function and attention^15,27^. In addition, Balzano and colleagues examined the cerebellum of patients with pre-cirrhotic steatosis caused by alcohol or metabolic syndrome postmortem histopathologically and were able to show that these patients displayed similar abnormalities^24^. Regarding the better PHES performance of patients with IALD, it could be postulated that the deficits in attention and working memory that are already present in the non-cirrhotic stage are merged with CogIm that arise in advanced liver disease^16,17^. Regarding the PHES results of the CRYO patients, it should be noted that in this group of patients more than 50% had a cryptogenic cause of liver cirrhosis. Steatotic causes may have been overlooked here. In most previous studies examining the impact of etiology on cognitive performance in patients with liver cirrhosis, etiologies are examined in less detail, often placing MASH among “Others”, which frequently includes cholestatic and autoimmune etiologies too. This may explain why these studies have mostly observed little or no effect of underlying etiology^3,12,23^. Noteworthy is a study by Sorrell and colleagues that investigated the results of the Repeatable Battery for the Assessment of Neuropsychological Status (RBANS) in patients on the LTx waiting list. The RBANS also covers a variety of cognitive domains and has age- and education-adjusted norm values. Here, in line with our results, patients with cholestatic disorders or HCV as underlying etiology performed better than patients with ALD, especially in terms of attention and immediate memory^19^.

In addition to the evaluation of CogIm in patients with liver cirrhosis, mHE tests are also used for risk stratification of the development of oHE. In this regard, the findings above raise the question to which extent etiology of liver cirrhosis has an impact upon the predictive value of these tests for the development of oHE. Our results suggest that mHE tests are less reliable for oHE risk stratification in patients with alcoholic and/or metabolic etiology, than in patients with viral, cholestatic or autoimmunologic etiology. Considering that in patients with ALD, MASH and MetALD the genesis of CogIm is not only caused by liver cirrhosis but additively by the metabolic syndrome or alcohol abuse, this would explain why the hepatological complication of an oHE can only be moderately predicted by abnormal neurocognitive test result. This additive effect was particularly evident in our ALD group. Here, the ideal cut-off of -8 calculated by us using maxstat to predict oHE may indicate that the “baseline” cognition in patients with liver cirrhosis due to ALD is lower than in patients with IALD and therefore a lower PHES score better predicts oHE in ALD patients. It should also be emphasized that patients with liver cirrhosis in whom CogIms are exclusively due to mHE are a rarity^8^. Due to the limited value of the other mHE tests in predicting oHE in our previous study, that included 50% of the patients in the present study, we focused exclusively on the PSE-Syndrome test for prediction^28^.

PSE-Syndrome test, but also Stroop test and ICT are complex tasks that combine a variety of cognitive abilities in varying degrees, e.g., motor speed and accuracy, visual perception, concentration, and attention. We can only speculate as to why the test results are influenced differently by the underlying etiology. One factor could be insufficient age- and education-adjusted cut-offs for evaluating the test results^21,22,28,29^. On the other hand, our results are consistent with the corresponding publications by the inventors, which show similar results indicating that the etiology of liver cirrhosis does not appear to influence the test result^21,29,30^. The above discussed mechanisms do not explain why patients with ALD performed significantly better in CRT. Since CRT primarily requires psychomotor speed and attention, it is surprising that patients with ALD showed the best results compared to other etiologies. Impairments of these cognitive abilities are frequently observed in patients with long-term alcohol abuse^31^. Yet another notable aspect of this study is the observation that - contrary to previous studies - the CFF test results are not worse in patients with ALD^20,32^.

Our study has some limitations: First of all, our findings are hypothesis-generating rather than definitive, given the number of subjects included in this study. Although we had a large, well-characterized total cohort, dividing it by etiology led to smaller subgroups, reducing the power of comparisons between etiologies. Second, it remains difficult to directly compare cirrhosis etiologies due to the heterogeneous nature of the underlying diseases. This applies in particular to the IALD group. This group consists of very different patterns of liver damage (cholestatic, HCV, autoimmune). We created this group because, on the one hand, individual etiologies were too rare in our cohort and, on the other hand, because previous studies on these etiologies in non-cirrhotic stages showed similar cognitive impairment and structural damage in qMRI^16,17^. Accordingly, we believe that these patients are more similar neurologically than they may appear hepatologically. Nevertheless, our pooling of these etiologies may mask real differences between them and complicates the clinical transferability of our results. Unfortunately, we also do not have brain imaging of the patients in our cohort. Another limitation is that due to the number of subjects included and the diversity of the cohort we could not control or adjust for many factors with impact upon cognition such as electrolyte imbalances or internal concomitant disorders. For example, 22% of our IALD patients have diabetes mellitus compared to 64% of the MASH group. Since diabetes control was good in the majority of the patients this should not have influenced the results. Moreover, the rate of patients with diabetes was similar in the two groups that differed most – ALD and IALD. Also, despite our adjustment, residual confounding cannot be completely ruled out, as, for example, only 51% of patients with CRYO had German as their native language, whereas this proportion was 98% among patients with MetALD. However, this is partly due to the genesis of the etiology of liver cirrhosis (such as diabetes mellitus and MASH or MetALD), meaning that even if the cohort were enlarged, this problem would still remain. In any case, the pooled IALD category and the residual baseline imbalances limit direct clinical translation, and external validation in etiology-specific cohorts is needed.

Additionally, while maxstat were generated to explore the relationship between mHE test results and oHE development, the thereby achieved cut-off values should not be used as definitive cut-offs for prediction. The cut-off of < -4 has been established as marker of abnormal test results using a German norm cohort. The maxstat generated cut-offs were intended to illustrate the variability in cognitive test performance across different etiologies, and the absence of a clear correlation between poor test results and higher oHE risk highlights the intricacy of using mHE tests as predictive tools. Establishing an etiology-specific cut-off of PHES or another mHE test is neither useful for the diagnosis of mHE nor for the prediction of oHE, as mHE tests can only detect a CogIm, which is multifaceted in patients with liver cirrhosis.

In conclusion, etiology seems to have impact upon results on the widely used reference standard, the PSE-Syndrome test. Our findings suggest that this test may be particularly valuable for predicting oHE in etiologies that have less impact on cognition, such as non-steatotic liver diseases. In contrast, steatotic conditions like ALD, MASH and MetALD may require alternative approaches for oHE risk stratification. Not every CogIm in patients with liver cirrhosis is a synonym for mHE, especially not in SLD patients.

## Methods

### Study cohort

We used two different study cohorts (cohort A and cohort B, Fig. 3). For cohort A, 292 patients with liver cirrhosis were recruited within a prospective study protocol at Hannover Medical School between August 2021 and July 2023. Outpatients were examined at the hepatology outpatient clinic of Hannover Medical School. Inpatients were usually examined during their stay for TIPS or LTx evaluation, or after elective admission for a procedure (e.g., endoscopy or measurement of hepatic venous pressure gradient). Part of cohort A (*n* = 73) had already been used to compare the diagnostic value of the six most commonly used methods for diagnosing mHE^28^. Cohort B consists of patients who were prospectively recruited between August 2019 and February 2024 as part of the observational Hannover TIPS study (clinical trials number NCT04801290) and who underwent an mHE assessment, consisting of at least the PHES (*n* = 222). For some of the patients, we were also able to access test results in CFF (*n* = 183). We excluded patients from cohort B who were already part of cohort A (*n* = 112). Part of this cohort (*n* = 84) has already been used to determine the predictive value of mHE tests for predicting oHE post-TIPS^33^.

The predefined exclusion criteria included insufficient evidence of liver cirrhosis, recent malignant tumor diagnosis (except for hepatocellular carcinoma within Milan criteria), age under 18 years, history of organ transplantation, language barriers, severe neurological comorbidities, kidney impairment with an estimated glomerular filtration rate (eGFR) below 30 ml/min/1.73 m², significant visual or hearing impairment, and lack of written informed consent.

After applying these criteria, 45 patients were excluded: five due to insufficient evidence of cirrhosis, nine with severe neurological comorbidities, ten with kidney impairment, and 16 with hepatocellular carcinoma outside the Milan criteria or other malignant diseases. Five patients declined the mHE assessment after study inclusion. Additionally, patients without PHES (*n* = 2) and with presence of HE grade 1 or 2 (*n* = 43) were excluded. In consequence, data from 312 patients were available for further analyses (PHES *n* = 312, CRT *n* = 230, Stroop *n* = 223, ANT *n* = 230, ICT *n* = 216, CFF *n* = 272, Fig. 3). We use the terms “no HE” (normal mHE test result, no HE ≥ grade 1), “mHE” (abnormal test result in an mHE test without the presence of HE ≥ grade 1), and “overt HE” (HE ≥ grade 2) in our manuscript.

Liver cirrhosis was diagnosed by laboratory, histopathological, sonographic, radiological, or liver-stiffness parameters. All patients were evaluated by trained staff. If patients were unable to complete a test due to CogIm, the test result was classified as abnormal.

### Minimal hepatic encephalopathy tests

The patients performed an extended mHE assessment consisting of PSE-Syndrome Test yielding the PHES (cut-off < -4)^34,35^, CRT (cut-off Index < 1.9)^36,37^, EncephalApp, providing the Stroop test (German age-, and education-adjusted cut-off)^38,39^, ICT (German age- and education-adjusted cut-offs)^22,32^, CFF (German age-adjusted cut-offs)^20,32^, and ANT^29^. For ANT, results worse than the 10th percentile (z-Score < -1.282) according to the age-, sex- and education-adjusted norm values of the Basel version of the “Consortium to Establish a Registry for Alzheimer’s Disease, Neuropsychological test battery” (CERAD-NP) were considered abnormal^40,41^. Test sequence was changed with each patient to control for a decline in concentration during the testing.

### Handling different etiologies of liver cirrhosis

In order to be able to assign the etiology as accurately as possible, alcohol consumption was exactly inquired. This resulted in an exact group assignment for almost all patients. If there was an additional risky alcohol consumption in a patient (> 60 g/day for men, > 50 g/day for women)^42^ with HCV infection, the patient was assigned to the IALD group (*n* = 4). We opted for this method in order to assign these patients to the group with less CogIm according to previous assumptions, so as not to overestimate the effect of the etiology in case of doubt^31^. The situation was similar for patients with hepatitis B virus (HBV) plus HCV infection (*n* = 1). This patient was also assigned to the IALD group. In this case, we chose this group assignment because no cognitive deficits were found in patients with HBV in the non-cirrhotic stage, while HCV infection is well-known to carry the risk of cognitive dysfunction irrespective of the grade of liver disease^16^. For patients with ALD, abstinence was defined as at least six months without alcohol consumption. Ethyl glucuronide was measured once in the patients’ urine. If this corresponded with the anamnestic information, further abstinence was assumed based on anamnestic data.

### Follow-up data and predictive value

To examine the extent to which liver cirrhosis etiology influences the predictive value of a mHE test, the patients were followed up for one year with a focus on the development of oHE, LTx or death (Fig. 3).

### Statistical methods

Quantitative data are presented as median with interquartile range (IQR), while categorical variables are reported as absolute and relative frequencies. Continuous data were analyzed using Kruskal-Wallis test. Categorical data were analyzed with χ^2^ test.

For determining the impact of cirrhosis etiology on different mHE tests, univariable and multivariable linear and binary logistic regression analyses were conducted. The most frequent etiology, ALD, was used as reference group. The primary objective of our analyses was not prediction, but rather to establish a causal relationship between the etiology of liver cirrhosis and the score results. For this reason, a data-driven analysis is insufficient per se; instead, the identification of the adjustment set should be performed using causal diagrams (i.e. directed acyclic graphs (DAGs)). Therefore, to estimate the total effect of etiology on the respected test scores, sex, age, native language, and the presence of diabetes mellitus were identified as the minimum sufficient adjustment group for multivariable analyses using a DAG model with DAGitty V3.0 (Model A, Suppl. Figure 3)^43^. This was done to represent the total causal effect of the etiology of liver cirrhosis on the respective test results. As a sensitivity analysis, a second data-driven multivariable model was established. Here, we adjusted for variables that were statistically significantly different between groups (*p* < 0.05, Model B, Suppl. Table 6). Variables included in MELD were excluded to avoid multicollinearity. Multicollinearity was excluded using the variance inflation factor (VIF). All VIF values were < 5. Heteroscedasticity was precluded from multivariable linear regression. Regression coefficients and odds ratios are reported with respected 95% confidence intervals (CI).

Furthermore, we utilized maximally selected rank statistics to determine whether patients with different etiologies show variations in test scores when developing oHE during one-year follow-up^44^. For this purpose, patients were censored after LTx, death or loss to follow-up. Here, the calculated ideal cut-offs for the prediction of oHE were compared between the different etiologies and interpreted as suitable and unsuitable based on the calculated *p*-value.

Fine & Gray’s competing risk analyses^45^ were conducted to assess the risk for oHE development during a 365-day follow-up associated with an abnormal PHES in relation to the underlying etiology. Here, death and LTx were treated as competing events.

All analyses were performed using IBM SPSS Statistics (version 29, SPSS Inc., Chicago, Illinois, USA), GraphPad Prism for Windows (version 10.2.1, GraphPad Software, Boston, Massachusetts USA) and R (version 4.3.0). In R, we utilized the packages “RCmdr” (version 2.9-1)^46,47^, “RcmdrPlugin.EZR” (version 1.62)^48^, “tableone” (version 0.13.2)^49^, and “maxstat” (version 0.7–25)^50^.

*P-*values < 0.05 were considered statistically significant and are assessed descriptively.

## Supplementary Information

Below is the link to the electronic supplementary material.

Supplementary Material 1

## Data Availability

The data that support the findings of this study are available from the corresponding author upon reasonable request.
