# Supplementary material for: Impact of liver cirrhosis etiology on results of diagnostic tests for minimal hepatic encephalopathy
Source: Sci Rep. 2026 Apr 22;16:13154. doi: 10.1038/s41598-026-49607-8 (PMC13102944; doi:10.1038/s41598-026-49607-8)
Supplement: Supplementary file 1 — Supplementary Material 1 [file 41598_2026_49607_MOESM1_ESM.pdf]

Supplementary material

Supplementary table 1. Breakdown of infectious or autoimmune liver disease group (n=63) 2

Supplementary table 2. Comparison of subgroups with and without TIPS at baseline 3

Supplementary table 3a. Univariable linear regression analyses for mHE test results at baseline 5

Supplementary table 3b. Multivariable linear regression analyses for mHE test results at baseline, Model B 7

Supplementary table 4a. Univariable binary logistic regression analyses for abnormal mHE test results at baseline 9

Supplementary table 4b. Multivariable binary logistic regression analyses for abnormal mHE test results at baseline, Model B 11

Supplementary table 5. Number of events (overt hepatic encephalopathy and death or liver transplantation) by etiology during 1 year follow-up 13

Supplementary table 6. Variables adjusted for in Model B 14

Supplementary figure 1. Box plots of mHE test results grouped by etiology.......... 15

Supplementary figure 2. Standardized log-rank statistics for the ideal cut-off value of PHES for predicting overt hepatic encephalopathy in 1 year.......... 16

Supplementary figure 3. Directed acyclic graphs to visualize the associations between etiology and minimal hepatic encephalopathy test results 17

Supplementary table 1. Breakdown of infectious or autoimmune liver disease group (n=63)

Etiology	Number of patients
**HCV**	16 (25%)
**AIH**	14 (22%)
**PSC**	10 (16%)
**PBC**	10 (16%)
**PSC AIH overlap**	9 (15%)
**Other cholangitis**	4 (6%)

All variables are presented as frequencies and percentages.
Abbreviations: AIH: Autoimmune hepatitis, HCV: Hepatitis C virus, PBC: primary biliary cholangitis, PSC: primary sclerosing cholangitis

Supplementary table 2. Comparison of subgroups with and without TIPS at baseline

	All patients	No TIPS	TIPS	Missing %	p-value
**Number of patients**	312	268	44		
**Age**	59 [51, 65]	58.5 [51, 65]	60.5 [50, 66.25]		0.718
**Sex male**	216 (69%)	189 (71%)	27 (61%)		0.297
**Child-Pugh-Score**	7 [6, 9]	8 [6, 9]	6 [5, 7.25]	2.2	**0.006**
**MELD**	12 [9, 16]	12 [9, 16]	11 [9, 13]	0.3	0.097
**Inpatients**	241 (77%)	218 (81%)	23 (52%)		**<0.001**
**Years of school education**	10 [9, 11]	10 [9, 11]	10 [9, 10]		0.292
**Diabetes mellitus**	93 (30%)	81 (30%)	12 (27%)		0.827
**Diabetes controlled**	68 (73%)	58 (72%)	10 (83%)		0.613
**Cardiovascular disease**	126 (40%)	109 (41%)	17 (39%)		0.929
**German as native language**	259 (83%)	224 (84%)	35 (80%)		0.657
**Any HE prophylaxis**	194 (62%)	159 (59%)	35 (80%)		**0.017**
**Lactulose intake**	165 (53%)	137 (51%)	28 (64%)		0.168
**Rifaximin intake**	88 (28%)	65 (24%)	23 (52%)		**<0.001**
**LOLA intake**	44 (14%)	34 (13%)	10 (23%)		0.124
**Change in HE prophylaxis during FU**	117 (38%)	104 (39%)	13 (30%)		0.313
**Any HE prophylaxis EoF**	223 (72%)	187 (70%)	36 (82%)		0.144
**Lactulose intake EoF**	207 (66%)	174 (65%)	33 (75%)		0.255
**Rifaximin intake EoF**	131 (42)	102 (38)	29 (66)		**0.001**
**LOLA intake EoF**	52 (17%)	43 (16%)	9 (21%)		0.611
**Previous oHE episode**	92 (30%)	76 (28%)	16 (36%)		0.368
**Liver biopsy**	133 (43%)	115 (43%)	18 (41%)		0.933
**Sodium (mmol/l)**	137 [134, 139]	136 [134, 139]	138 [136, 140]		**0.004**
**Creatinine (µmol/l)**	90 [74, 117.25]	91.5 [74.75, 120.5]	86 [71, 103.25]		0.146
**CHE (kU/l)**	3.05 [2, 4.37]	2.92 [1.92, 4.16]	3.54 [2.5, 5.15]	3.5	**0.002**
**Bilirubin (µmol/l)**	21 [13, 45]	21 [13, 46.25]	23.5 [15, 38]		0.599
**Albumin (g/dl)**	33 [28, 38]	33 [28, 38]	34 [29.25, 38]	2.2	0.352
**White blood cells (tsd/µl)**	5.1 [3.6, 7]	5.1 [3.5, 6.9]	5.3 [4, 7.65]		0.248
**Platelets (tsd/µl)**	101 [62.5, 155.5]	100 [61, 154.5]	114.5 [83.25, 171.5]	0.3	0.115
**Hemoglobin (g/dl)**	10.7 [9.28, 12.9]	10.6 [8.97, 12.8]	11.65 [9.97, 13.43]		**0.028**
**INR**	1.24 [1.11, 1.37]	1.24 [1.12, 1.38]	1.21 [1.08, 1.35]	0.3	0.32
**PHES**	-4 [-7, -1.75]	-4 [-7, -2.]	-3 [-6, -1]		0.5
**PHES abnormal (<-4)**	126 (40%)	109 (41%)	17 (39%)		0.929
**CRT Index**	1.91 [1.46, 2.32]	1.92 [1.5, 2.34]	1.86 [1.33, 2.31]	26.3	0.356
**CRT abnormal**	113 (49%)	91 (49%)	22 (51%)	26.3	0.899
**Stroop Off+OnTime (sec)**	185.53 [161.87, 208.28]	185.96 [162.12, 208.09]	185.14 [158.68, 213.52]	30.4	0.978
**Stroop OffTime (sec)**	85.67 [76.28, 96.43]	86.40 [77.14, 95.63]	83.34 [73.06, 99.61]	30.4	0.742
**Stroop OnTime (sec)**	100.47 [85.22, 113.14]	100.26 [85.22, 112.68]	102.06 [85.73, 115.57]	30.4	0.744
**Stroop abnormal**	111 (50%)	90 (50%)	21 (51%)	28.5	0.975
**ANT (animals/minute)**	22 [19, 27]	22 [19, 27]	23.5 [18.25, 30]	26.3	0.197
**ANT z-Score**	-0.23 [-1.06, 0.6]	-0.26 [-1.1, 0.54]	0.17 [-0.64, 0.75]	26.3	0.06
**ANT abnormal**	45 (29%)	41 (22%)	4 (10%)	26.3	0.11
**ICT weighted lures**	17.86 [9.89, 30.26]	15.89 [8.56, 28.23]	24.32 [13.90, 35.57]	34.6	**0.007**
**ICT abnormal**	67 (31)	49 (28)	18 (46)	30.8	**0.039**
**CFF (in Hz)**	41.8 [38.5, 46.75]	41.8 [38.6, 46.64]	41.7 [37.77, 46.60]	12.5	0.986
**CFF abnormal**	60 (22%)	49 (21%)	11 (26%)	12.5	0.674

All continuous variables are presented as median and interquartile range, dichotomous values are presented as absolute and relative frequencies, Kruskal-Wallis test for continuous variables, χ-2 for dichotomous values, values of p <0.05 are highlighted in bold font.

Abbreviations: ALD: alcohol-related liver disease, ANT: Animal Naming Test, CFF: Critical Flicker Frequency, CHE: cholinesterase, CRT: Continuous Reaction Time Test, CRYO: cryptogenic/other, EoF: end of follow-up HE: hepatic

encephalopathy, IALD: infectious/autoimmune liver disease, ICT: Inhibitory Control Test, INR: international normalized ratio, MASH: metabolic dysfunction-associated steatohepatitis, MELD: model for end-stage liver disease, MetALD metabolic and alcohol-associated liver disease, LOLA: L-ornithine-L-aspartate, oHE: overt hepatic encephalopathy, PHES: Portosystemic Hepatic Encephalopathy Score, TIPS: transjugular intrahepatic portosystemic shunt

Supplementary table 3a. Univariable linear regression analyses for mHE test results at baseline

	PHES		CRT Index		Stroop Off+On Time		ANT's z-score		ICT's Weighted Lures		CFF in Hz	
	**RCβ (95% CI)**	**p-value**	**RCβ (95% CI)**	**p-value**	**RCβ (95% CI)**	**p-value**	**RCβ (95% CI)**	**p-value**	**RCβ (95% CI)**	**p-value**	**RCβ (95% CI)**	**p-value**
**Previous oHE-episode**	-0.96 (-1.923 - 0.022)	0.055	**-0.292 (-0.481 - -0.103)**	**0.003**	8.109 (-5.083 – 21.301)	0.227	-0.237 (-0.601 - 0.127)	0.201	3.565 (-2.22 9.35)	0.226	-1.374 (-2.997 – 0.249)	0.097
**Age**	**-0.047 (-0.086 - -0.008)**	**0.017**	0.003 (-0.004 – 0.011)	0.383	**1.681 (1.233 – 2.129)**	**<0.001**	0.013 (-0.001 – 0.026)	0.72	**0.361 (0.143 – 0.579)**	**0.001**	-0.012 (-0.078 – 0.054)	0.726
**Years of school education**	**0.374 (0.108 - 0.640)**	**0.006**	0 (-0.053 – 0.053)	0.995	**-6.08 (-9.587 - -2.573)**	**<0.001**	0.066 (-0.036 – 0.169)	0.204	**-1.918 (-3.581 - -0.255)**	**0.024**	0.01 (-0.437 – 0.456)	0.966
**Sex male**	-0.721 (-1.694 – 0.252)	0.146	**0.258 (0.071 – 0.444)**	**0.007**	5.274 (-7.522 – 18.069)	0.417	0.245 (-0.113 – 0.603)	0.178	-2.143 (-7.833 – 3.547)	0.459	-0.485 (-2.098 - 1.129	0.555
**Diabetes mellitus**	-0.827 (-1.808 - 0.154	0.098	-0.11 (-0.306 – 0.087)	0.274	12.885 (-0.698 – 26.469)	0.063	**0.475 (0.108 – 0.843)**	**0.011**	5.408 (-0.548 – 11.364)	0.075	-0.719 (-2.366 – 0.927)	0.391
**Diabetes controlled**	0.928 (-1.089 - 2.944)	0.363	-0.083 (-0.469 – 0.303)	0.667	-3.374 (-37.737 – 30.989)	0.845	0.457 (-0.271 – 1.185)	0.214	-3.184 (-19.401 – 13.033)	0.695	-2.398 (-5.472 – 0.676)	0.124
**CV disease**	-0.293 (-1.211 - 0.624)	0.53	-0.029 (-0.209 – 0.15)	0.747	**15.319 (3.284 – 27.355)**	**0.013**	0.147 (-0.193 – 0.486)	0.396	**6.393 (1.021 – 11.765)**	**0.02**	0.031 (-1.484 – 1.545)	0.968
**TIPS**	0.43 (-0.864 – 1.724)	0.513	-0.123 (-0.346 – 0.1)	0.279	0.076 (-15.192 – 15.344	0.992	0.314 (-0.112 – 0.74)	0.148	5.35 (-1.441 – 12.141)	0.122	0.044 (-2.008 – 2.097)	0.966
**Inpatients**	**-1.794 (-2.85 - -0.738)**	**<0.001**	-0.001 (-0.191 – 0.188)	0.99	4.35 (-8.451 – 17.152)	0.504	-0.015 (-0.376 – 0.346)	0.936	3.002 (-2.733 – 8.737)	0.303	-1.588 (-3.324 – 0.148)	0.073
**German as native language**	0.592 (-0.606 – 1.79)	0.332	0.06 (-0.163 – 0.284)	0.596	-6 (-21.398 – 9.398)	0.443	**1.148 (0.747 – 1.549)**	**<0.001**	-2.419 (-9.394 – 4.555)	0.495	0.546 (-1.487 – 2.578)	0.597
**MELD**	**-0.18 (-0.268 – -0.093)**	**<0.001**	-0.016 (-0.033 – 0.001)	0.067	0.555 (-0.587- 1.697)	0.339	**-0.045 (-0.077 - -0.013)**	**0.006**	-0.005 (-0.53 – 0.519)	0.985	-0.061 (-0.214 – 0.092)	0.433
**Child-Pugh-Score**	**-0.633 (-0.866 - -0.399)**	**<0.001**	-0.039 (-0.086 – 0.008)	0.1	1.366 (-1.851 – 4.584)	0.404	-0.087 (-0.175 – 0.002)	0.054	0.197 (-1.206 – 1.599)	0.782	**-0.456 (-0.873 – -0.04)**	**0.032**
**HE medication**												
**Any HE prophylaxis**	**-2.106 (-3.005 - -1.208)**	**<0.001**	**-0.255 (-0.429 - -0.08)**	**0.004**	**14.469 (2.611- 26.328)**	**0.017**	-0.189 (-0.524 – 0.146)	0.679	2.921 (-2.378 – 8.221)	0.278	-1.021 (-2.548 – 0.506)	0.189
**Lactulose intake**	**-2.023 (-2.897 - -1.149)**	**<0.001**	**-0.228 (-0.4 - -0.056**	**0.01**	**15.095 (3.408- 26.782)**	**0.012**	-0.157 (-0.488 – 0.174)	0.35	2.161 (-3.121 – 7.443)	0.421	-0.282 (-1.777 – 1.213)	0.711

**Rifaximin intake**	**-1.274 (-2.265 - -0.283)**	**0.012**	**-0.254 (-0.452 – -0.056)**	**0.012**	4.12 (-9.649 – 17.889)	0.556	-0.026 (-0.406 - 0.355)	0.895	**6.181 (0.122 – 12.241)**	**0.046**	-0.193 (-1.848 – 1.461)	0.818
**L-Ornithine L-Aspartate intake**	**-1.581 (-2.863 - -0.298)**	**0.016**	-0.126 (-0.384 – 0.133)	0.339	**30.788 (13.62 – 47.956)**	**<0.001**	-0.453 (-0.94 - 0.035)	0.069	2.36 (-5.527 – 10.247)	0.556	-1.103 (-3.213 – 1.007)	0.304
**Lab values**												
**Sodium (mmol/l)**	**0.185 (0.081 - 0.289)**	**<0.001**	**0.025 (0.004 – 0.047)**	**0.019**	**-1.766 (-3.243 - -0.289)**	**0.019**	0.008 (-0.033 – 0.049)	0.701	-0.143 (-0.803 – 0.518)	0.671	0.144 (-0.045 – 0.332)	0.135
**Creatinine (µmol/l)**	**-0.031 (-0.043 - -0.02)**	**<0.001**	-0.002 (-0.005 – 0)	0.069	**0.201 (0.023 – 0.378)**	**0.027**	-0.002 (-0.007 – 0.003)	0.417	0.044 (-0.035 – 0.123)	0.27	-0.02 (-0.04 – 0)	0.056
**CHE (kU/l)**	**0.707 (0.451 - 0.962)**	**<0.001**	0.041 (-0.1 – 0.093)	0.117	-3.431 (-6.901 – 0.038)	0.053	**0.158 (0.064 – 0.252)**	**0.001**	0.531 (-1.031 – 2.094)	0.503	**0.686 (0.251 – 1.122)**	**0.002**
**Bilirubin (µmol/l)**	-0.001 (-0.008 – 0.005)	0.667	0 (-0.001 – 0.001)	0.975	-0.008 (-0.077 – 0.062)	0.83	-0.001 (-0.003 - 0.001)	0.428	-0.021 (-0.053 – 0.012)	0.207	-0.001 (0.012 – 0.009)	0.826
**Albumin (g/l)**	**0.07 (0.007 – 0.132)**	**0.029**	**0.13 (0 - 0.025)**	**0.047**	-0.275 (-1.131 - 0.58)	0.526	**0.035 (0.012 – 0.058)**	**0.003**	-0.022 (-0.396 – 0.352)	0.906	**0.165 (0.059 – 0.27)**	**0.002**
**White blood cells (tsd/µl)**	-0.123 (-0.274 – 0.029)	0.111	-0.011 (-0.04 – 0.017)	0.432	-0.047 (-1.963 – 1.868)	0.961	0.037 (-0.017 – 0.091)	0.179	-0.047 (-0.899 – 0.805)	0.914	-0.047 (-0.295 – 0.202)	0.712
**Platelets (tsd/µl)**	-0.003 (-0.009 – 0.003)	0.334	0 (-0.002 – 0.001)	0.45	-0.023 (-0.103 – 0.057)	0.578	0.002 (0-0.004)	0.092	-0.013 (-0.048 – 0.023)	0.481	-0.006 (-0.016 – 0.004)	0.226
**Hemoglobin (g/dl)**	**0.447 (0.263 – 0.632)**	**<0.001**	**0.044 (0.008 – 0.081)**	**0.017**	-2.229 (-4.705 – 0.247)	0.077	**0.119 (0.052 – 0.187)**	**<0.001**	0.216 (-0.892 – 1.323)	0.701	**0.461 (0.14 – 0.783)**	**0.005**
**INR**	**-2.307 (-4.064 - -0.55)**	**0.01**	-0.142 (-0.471 – 0.187)	0.396	9.499 (-12.696 – 31.693)	0.4	**-0.747 (-1.365 - -0.129)**	**0.018**	0.437 (-9.379 – 10.253)	0.93	-0.854 (-3.809 – 2.101)	0.57
**Etiology**												
**MASH**	0.401 (-0.909 – 1.71)	0.548	-0.012 (-0.278 – 0.254)	0.929	9.757 (-7.734 – 27.249)	0.273	0.065 (-0.435 - 0.564)	0.798	0.665 (-7.173 – 8.503)	0.867	1.076 (-1.143 – 3.295)	0.341
**MetALD**	-0.329 (-1.761 – 1.104)	0.652	-0.115 (-0.423 – 0.193)	0.464	16.431 (-4.659 – 37.521)	0.126	-0.181 (-0.76 – 0.398)	0.539	1.314 (-7.845 – 10.474)	0.778	-1.389 (-3.813 – 1.036)	0.26
**IALD**	**1.944 (0.709 – 3.18)**	**0.002**	-0.059 (-0.3 – 0.182)	0.631	-15.196 (-31.054 – 0.663)	0.06	-0.257 (-0.711 – 0.196)	0.264	-3.592 (-10.846 – 3.662)	0.33	0.482 (-1.568 – 2.352	0.644
**CRYO**	0.678 (-0.688 – 2.044)	0.329	-0.126 (-0.373 – 0.122)	0.32	4.5 (-12.064- 21.064)	0.593	**-0.495 (-0.961 - -0.029**	**0.038**	4.518 (-3.028 – 12.063)	0.239	1.075 (-1.219 – 3.37)	0.357

Univariable linear regression analyses. All variables are shown as regression coefficient ß with 95% confidence interval in parentheses, p-values <0.05 are highlighted in bold font

Abbreviations: ALD: alcohol-related liver disease, ANT: Animal Naming Test, CFF: Critical Flicker Frequency, CHE: cholinesterase, CRT: Continuous Reaction Time Test, CRYO: cryptogenic/other, CV: cardiovascular, HE: hepatic encephalopathy, IALD: infectious/autoimmune liver disease, ICT: Inhibitory Control Test, INR: international normalized ratio, MASH: metabolic dysfunction-associated steatohepatitis, MELD: model for end-stage liver disease, MetALD metabolic and alcohol-associated liver disease, mHE: minimal hepatic encephalopathy, LOLA: L-ornithine-L-aspartate, oHE: overt hepatic encephalopathy, PHES: Portosystemic Hepatic Encephalopathy Score, RCβ: regression coefficient β, TIPS: transjugular intrahepatic portosystemic shunt

Supplementary table 3b. Multivariable linear regression analyses for mHE test results at baseline, Model B

	PHES		CRT Index		Stroop Off+On Time		ANT's z-score		ICT's Weighted Lures		CFF in Hz	
	**aRCβ (95% CI)**	**p-value**	**aRCβ (95% CI)**	**p-value**	**aRCβ (95% CI)**	**p-value**	**aRCβ (95% CI)**	**p-value**	**aRCβ (95% CI)**	**p-value**	**aRCβ (95% CI)**	**p-value**
**Previous oHE-episode**			**-0.283 (-0.471 - -0.095)**	**0.003**								
**Age**	**-0.049 (-0.088 - -0.01)**	**0.013**			**1.484 (1.012 – 1.957)**	**<0.001**			**0.31 (0.08 – 0.539)**	**0.009**		
**Years of school education**	**0.349 (0.096 - 0.603)**	**0.007**			**-4.372 (-7.576 - -1.169)**	**0.008**			-1.462 (-3.11 - - 0.187)	0.082		
**Sex male**			**0.271 (0.082 – 0.46)**	**0.005**								
**Diabetes mellitus**							**0.428 (0.074 – 0.781)**	**0.018**				
**CV disease**					3.507 (-8.102 – 15.115)	0.552			5.197 (-0.578 – 10.972)	0.077		
**Inpatients**	-0.692 (-1.788 - 0.404)	0.215										
**German as native language**							**1.234 (0.818 – 1.65)**	**<0.001**				
**MELD**	**-0.138 (-0.23 – -0.046)**	**0.003**					-0.02 (-0.052 - -0.012)	0.22				
**Child-Pugh-Score**											-0.18 (-0.667 – 0.306)	0.466
**Lab values**												
**Sodium (mmol/l)**	0.088 (-0.015 - 0.191)	0.095	0.019 (-0.004 – 0.041)	0.1	**-1.588 (-2.963 - -0.212)**	**0.024**						
**Creatinine (µmol/l)**					0.074 (-0.094 – 0.241)	0.386						
**Albumin (g/l)**			0.007 (-0.008 - 0.022)	0.365								
**Hemoglobin (g/dl)**	**0.265 (0.06 – 0.469)**	**0.012**	0.02 (-0.024 – 0.065)	0.366			**0.128 (0.06 – 0.197)**	**<0.001**			0.367 (-0.16 – 0.75)	0.061
**Etiology**												

**MASH**	0.614 (-0.632 – 1.859)	0.333	-0.034 (-0.291 – 0.223)	0.796	1.644 (-14.607 – 17.895)	0.842	-0.203 (-0.674 - 0.268)	0.397	-3.321 (-11.255 – 4.614)	0.41	0.995 (-1.25 – 3.239)	0.384
**MetALD**	0.215 (-1.142 – 1.573)	0.755	-0.125 (-0.43 – 0.179)	0.418	3.223 (-16.282 – 22.729)	0.745	-0.276 (-0.802 – 0.25)	0.302	-2.946 (-12.208 – 6.317)	0.531	-1.187 (-3.608 – 1.234)	0.26
**IALD**	**1.765 (0.592 – 2.938)**	**0.003**	-0.038 (-0.275 – 0.199)	0.752	-6.874 (-21.218 – 7.47)	0.346	-0.214 (-0.624 – 0.196)	0.305	-2.336 (-9.396 – 4.724)	0.515	0.296 (-1.756 – 2.347)	0.777
**CRYO**	0.181 (-1.148 – 1.51)	0.789	-0.159 (-0.402 – 0.084)	0.198	4.639 (-10.289- 19.567)	0.541	-0.176 (-0.631 - 0.28)	0.338	4.372 (-2.931 – 11.675)	0.239	0.438 (-1.918 – 2.794)	0.715

Multivariable linear regression analyses with all variables with p <0.05 in univariable analysis. Variables included in MELD and HE prophylaxis were excluded due to multicollinearity. All variables are shown as adjusted regression coefficient ß with 95% confidence interval in parentheses, p-values <0.05 are highlighted in bold font

Abbreviations: ALD: alcohol-related liver disease, ANT: Animal Naming Test, aRCβ: adjusted regression coefficient β, CFF: Critical Flicker Frequency, CHE: cholinesterase, CRT: Continuous Reaction Time Test, CRYO: cryptogenic/other, CV: cardiovascular, HE: hepatic encephalopathy, IALD: infectious/autoimmune liver disease, ICT: Inhibitory Control Test, INR: international normalized ratio, MASH: metabolic dysfunction-associated steatohepatitis, MELD: model for end-stage liver disease, MetALD metabolic and alcohol-associated liver disease, mHE: minimal hepatic encephalopathy, oHE: overt hepatic encephalopathy, PHES: Portosystemic Hepatic Encephalopathy Score

Supplementary table 4a. Univariable binary logistic regression analyses for abnormal mHE test results at baseline

	**PHES abnormal**		**CRT abnormal**		**Stroop abnormal**		**ANT's z-Score abnormal**		**ICT abnormal**		**CFF adjusted abnormal**	
	**OR (95% CI)**	**p-value**	**OR (95% CI)**	**p-value**	**OR (95% CI)**	**p-value**	**OR (95% CI)**	**p-value**	**OR (95% CI)**	**p-value**	**OR (95% CI)**	**p-value**
**Previous oHE-episode**	**1.683 (1.029-2.752)**	**0.038**	**2.511 (1.387-4.544)**	**0.002**	1.156 (0.645-2.073)	0.625	1.155 (0.569-2.344)	0.690	0.945 (0.499-1.787)	0.861	1.782 (0.98-3.24)	0.058
**Age**	**1.031 (1.01-1.053)**	**0.004**	1.002 (0.980-1.024)	0.885	1.019 (0.996-1.042)	0.112	**0.963 (0.938-0.99)**	**0.006**	1.026 (0.999-1.053)	0.057	1.015 (0.989-1.042)	0.269
**Years of school education**	0.878 (0.765-1.008)	0.064	0.992 (0.848-1.160)	0.915	0.905 (0.770-1.064)	0.225	1.034 (0.844-1.266)	0.747	0.849 (0.701-1.03)	0.096	0.866 (0.727-1.032)	0.107
**Sex male**	1.237 (0.755-2.025)	0.399	**0.532 (0.3-0.941)**	**0.03**	1.169 (0.661-2.070)	0.591	0.521 (0.266-1.022)	0.058	1.05 (0.56-1.967)	0.88	0.725 (0.397-1.324)	0.296
**Diabetes mellitus**	**1.741 (1.066-2.843)**	**0.027**	1.721 (0.951-3.114)	0.073	0.922 (0.507-1.678)	0.791	**0.354 (0.142-0.885)**	**0.026**	1.768 (0.939-3.33)	0.078	0.866 (0.455-1.648)	0.661
**Diabetes controlled**	0.524 (0.295-1.862)	0.524	0.650 (0.192-2.205)	0.489	0.528 (0.160-1.744)	0.295	0.302 (0.054-1.682)	0.172	0.486 (0.147-1.605)	0.237	0.938 (0.263-3.343)	0.921
**CV disease**	1.267 (0.801-2.004)	0.313	1.650 (0.965-2.821)	0.067	1.015 (0.592-1.740)	0.958	0.596 (0.293-1.21)	0.152	**1.865 (1.038-3.353)**	**0.037**	0.976 (0.546-1.745)	0.935
**TIPS**	0.891 (0.463-1.712)	0.728	1.105 (0.569-2.145)	0.768	1.073 (0.545-2.114)	0.838	0.377 (0.127-1.119)	0.079	**2.239 (1.1-4.556)**	**0.026**	1.27 (0.597-2.7)	0.535
**Inpatients**	**2.472 (1.368-4.468)**	**0.003**	0.951 (0.542-1.668)	0.861	1.712 (0.963-3.044)	0.067	1.412 (0.668-2.981)	0.366	1.562 (0.184-3.014)	0.184	1.061 (0.54-2.084)	0.863
**German as native language**	0.937 (0.536-1.779)	0.976	0.807 (0.415-1.568)	0.527	0.638 (0.316-1.285)	0.208	**0.146 (0.07-0.307)**	**<0.001**	0.582 (0.284-1.191)	0.138	0.616 (0.299-1.269)	0.189
**MELD**	**1.066 (1.018-1.116)**	**0.007**	1.046 (0.993-1.102)	0.089	1.031 (0.979-1.086)	0.247	1.056 (0.995-1.121)	0.071	1.027 (0.971-1.086)	0.354	1.011 (0.955-1.071)	0.706
**Child-Pugh-Score**	**1.268 (1.116-1.44)**	**<0.001**	1.098 (0.954-1.264)	0.192	1.116 (0.966-1.289)	0.136	1.079 (0.909-1.28)	0.387	1.059 (0.907-1.235)	0.47	**1.183 (1.009-1.443)**	**0.038**
**HE medication**												
**Any HE prophylaxis**	**3.198 (1.93-5.299)**	**<0.001**	**2.103 (1.299-3.596)**	**0.007**	1.460 (0.860-2.477)	0.161	0.681 (0.354-1.311)	0.251	1.3 (0.719-2.349)	0.385	**2.281 (1.198-4.344)**	**0.012**
**Lactulose intake**	**3.442 (2.13-5.562)**	**<0.001**	**1.811 (1.073-3.056)**	**0.026**	1.460 (0.860-2.477)	0.161	0.882 (0.458-1.698)	0.707	1.119 (0.628-1.994)	0.703	1.602 (0.895-2.868)	0.113
**Rifaximin intake**	**1.778 (1.08-2.926)**	**0.024**	**2.226 (1.204-4.113)**	**0.011**	1.485 (0.807-2.734)	0.204	0.584 (0.254-1.339)	0.204	1.719 (0.906-3.259)	0.097	0.985 (0.522-1.859)	0.963

**L-Ornithine L-Aspartate intake**	**2.106 (1.106-4.019)**	**0.024**	1.948 (0.882-4.304)	0.099	1.777 (0.798-3.960)	0.160	0.8 (0.288-2.22)	0.668	1.43 (0.634-3.222)	0.389	1.424 (0.665-3.052)	0.363
**Lab values**												
**Sodium (mmol/l)**	**0.928 (0.879-0.98)**	**0.007**	0.944 (0.884-1.008)	0.084	0.938 (0.876-1.004)	0.066	0.962 (0.89-1.04)	0.335	1.013 (0.942-1.09)	0.73	0.951 (0.885-1.021)	0.162
**Creatinine (µmol/l)**	**1.015 (1.009-1.022)**	**<0.001**	1.005 (0.997-1.013)	0.223	1.008 (0.999-1.016)	0.068	1.001 (0.991-1.010)	0.911	1.005 (0.997-1.013)	0.241	**1.01 (1.002-1.017)**	**0.011**
**CHE (kU/l)**	**0.77 (0.665-0.892)**	**<0.001**	0.966 (0.828-1.126)	0.656	0.871 (0.742-1.022)	0.091	0.911 (0.741-1.116)	0.370	0.972 (0.818-1.155)	0.747	**0.755 (0.62-0.919)**	**0.005**
**Bilirubin (µmol/l)**	1 (0.997-1.003)	0.969	1 (0.997-1.003)	0.939	1 (0.997-1.004)	0.791	1 (0.996-1.004)	0.965	1 (0.996-1.003)	0.819	1.002 (0.998-1.005)	0.398
**Albumin (g/l)**	0.975 (0.944-1.008)	0.137	0.974 (0.939-1.011)	0.172	0.977 (0.940-1.015)	0.227	0.966 (0.966-1.013)	0.158	0.976 (0.936-1.017)	0.252	**0.928 (0.887-0.970)**	**0.001**
**White blood cells (tsd/µl)**	1.015 (0.941-1.095)	0.692	0.992 (0.911-1.080)	0.852	1.003 (0.921-1.093)	0.940	0.952 (0.847-1.069)	0.405	0.964 (0.873-1.064)	0.465	1.044 (0.954-1.143)	0.346
**Platelets (tsd/µl)**	1.001 (0.998-1.004)	0.365	1.001 (0.997-1.004)	0.635	1 (0.997-1.004)	0.839	1 (0.995-1.004)	0.860	1 (0.997-1.004)	0.805	**1.004 (1.001-1.008)**	**0.013**
**Hemoglobin (g/dl)**	**0.812 (0.733-0.9)**	**<0.001**	**0.895 (0.801-1)**	**0.049**	**0.861 (0.768 – 0.966)**	**0.011**	0.959 (0.836-1.1)	0.55	0.977 (0.866-1.102)	0.701	0.897 (0.789-1.019)	0.095
**INR**	1.754 (0.721-4.269)	0.215	1.324 (0.496-3.537)	0.576	1.731 (0.628 – 4.769)	0.289	2.070 (0.671-6.385)	0.205	1.682 (0.586-4.825)	0.334	0.453 (0.124-1.653)	0.23
**Etiology**												
**MASH**	0.774 (0.398-1.504)	0.45	1.915 (0.859-4.271)	0.112	0.872 (0.395-1.925)	0.734	0.958 (0.31-2.960)	0.941	1.209 (0.513-2.854)	0.664	0.725 (0.295-1.777)	0.482
**MetALD**	1.005 (0.491-2.058)	0.988	1.906 (0.760-4.781)	0.169	1.827 (0.694–4.807)	0.222	0.821 (0.212-3.188)	0.776	0.818 (0.284-2.353)	0.71	1.129 (0.463-2.75)	0.79
**IALD**	**0.422 (0.218-0.818)**	**0.011**	1.753 (0.852-3.609)	0.128	0.503 (0.237–1.065)	0.073	1.327 (0.514-3.429)	0.559	0.773 (0.332-1.801)	0.55	1.062 (0.495-2.277)	0.878
**CRYO**	**0.477 (0.229-0.992)**	**0.047**	**2.372 (1.107-5.081)**	**0.026**	1.462 (0.674-3.171)	0.337	**3.538 (1.477-8.479)**	**0.005**	1.484 (0.665-3.309)	0.335	0.593 (0.221-1.591)	0.299

Univariable logistic regression analyses. All variables are shown as Odds Ratio with 95% confidence interval in parentheses, p-values <0.05 are highlighted in bold font

Abbreviations: ALD: alcohol-related liver disease, ANT: Animal Naming Test, CFF: Critical Flicker Frequency, CHE: cholinesterase, CRT: Continuous Reaction Time Test, CRYO: cryptogenic/other, CV: cardiovascular, HE: hepatic encephalopathy, IALD: infectious/autoimmune liver disease, ICT: Inhibitory Control Test, INR: international normalized ratio, MASH: metabolic dysfunction-associated steatohepatitis, MELD: model for end-stage liver disease, MetALD metabolic and alcohol-associated liver disease, mHE: minimal hepatic encephalopathy, LOLA: L-ornithine-L-aspartate, oHE: overt hepatic encephalopathy, OR: Odds Ratio, PHES: Portosystemic Hepatic Encephalopathy Score, TIPS: transjugular intrahepatic portosystemic shunt

Supplementary table 4b. Multivariable binary logistic regression analyses for abnormal mHE test results at baseline, Model B

	**PHES abnormal**		**CRT abnormal**		**Stroop abnormal**		**ANT's z-Score abnormal**		**ICT abnormal**		**CFF adjusted abnormal**	
	**aOR (95% CI)**	**p-value**	**aOR (95% CI)**	**p-value**	**aOR (95% CI)**	**p-value**	**aOR (95% CI)**	**p-value**	**aOR (95% CI)**	**p-value**	**aOR (95% CI)**	**p-value**
**Previous oHE-episode**	1.451 (0.84-2.506)	0.182	**2.678 (1.435-4.994)**	**0.002**								
**Age**	**1.04 (1.015-1.066)**	**0.002**					0.979 (0.949-1.01)	0.179				
**Sex male**			**0.519 (0.283-0.952)**	**0.034**								
**Diabetes mellitus**	**1.846 (1.037-3.284)**	**0.037**					0.392 (0.143-1.077)	0.069				
**CV disease**									**2.003 (1.05-3.823)**	**0.035**		
**TIPS**									**2.532 (1.195-5.362)**	**0.015**		
**Inpatients**	1.467 (0.794-3.093)	0.262										
**German as native language**							**0.194 (0.083-0.455)**	**<0.001**				
**MELD**	1.055 (0.998-1.115)	0.058										
**Child-Pugh-Score**											1.058 (0.9-1.243)	0.496
**Lab values**												
**Sodium (mmol/l)**	0.963 (0.907-1.022)	0.215										
**Creatinine (µmol/l)**											1.005 (0.996-1.014)	0.25
**Platelets (tsd/µl)**											1.001 (0.997-1.005)	0.677
**Hemoglobin (g/dl)**	**0.862 (0.763-0.972)**	**0.016**	**0.881 (0.78-0.994)**	**0.04**	**0.835 (0.739 – 0.943)**	**0.004**						
**Etiology**												

**MASH**	0.537 (0.246-1.173)	0.119	2.21 (0.952-5.131)	0.065	0.951 (0.423-2.136)	0.903	1.449 (0.421-4.988)	0.556	0.974 (0.386-2.458)	0.955	1.427 (0.576-3.533)	0.442
**MetALD**	0.642 (0.295-1.397)	0.264	2.096 (0.796-5.519)	0.134	1.784 (0.667-4.773)	0.249	1.268 (0.301-5.336)	0.746	0.735 (0.241-2.242)	0.589	0.815 (0.28-2.377)	0.708
**IALD**	**0.451 (0.22-0.922)**	**0.029**	1.816 (0.851-3.877)	0.123	0.477 (0.221-1.027)	0.059	1.294 (0.473-3.544)	0.616	0.833 (0.348-1.993)	0.681	0.801 (0.329-1.948)	0.625
**CRYO**	0.664 (0.285-1.454)	0.289	**3.202 (1.395-7.347)**	**0.006**	1.847 (0.821-4.156)	0.138	1.972 (0.715-5.434)	0.189	1.786 (0.766-4.166)	0.179	1.746 (0.76-4.014)	0.189

Multivariable logistic regression analyses with all variables with p <0.05 in univariable analysis. Variables included in MELD and HE prophylaxis were excluded due to multicollinearity. All variables are shown as adjusted Odds Ratio with 95% confidence interval in parentheses, p-values <0.05 are highlighted in bold font

Abbreviations: ALD: alcohol-related liver disease, ANT: Animal Naming Test, aOR: adjusted Odds Ratio, CFF: Critical Flicker Frequency, CHE: cholinesterase, CRT: Continuous Reaction Time Test, CRYO: cryptogenic/other, CV: cardiovascular, HE: hepatic encephalopathy, IALD: infectious/autoimmune liver disease, ICT: Inhibitory Control Test, INR: international normalized ratio, MASH: metabolic dysfunction-associated steatohepatitis, MELD: model for end-stage liver disease, MetALD metabolic and alcohol-associated liver disease, mHE: minimal hepatic encephalopathy, oHE: overt hepatic encephalopathy, PHES: Portosystemic Hepatic Encephalopathy Score, TIPS: transjugular intrahepatic portosystemic shunt

Supplementary table 5. Number of events (overt hepatic encephalopathy and death or liver transplantation) by etiology during 1 year follow-up

Etiology	Loss to FU	oHE development	Death	LTx	Death or LTx before oHE
**ALD**	9 (8%)	27 (24%)	23 (21%)	2 (2%)	17 (15%)
**MASH**	4 (8%)	17 (33%)	10 (19%)	6 (12%)	9 (17%)
**MetALD**	6 (15%)	11 (26%)	7 (17%)	3 (7%)	5 (12%)
**IALD**	3 (5%)	18 (29%)	10 (16%)	13 (21%)	12 (19%)
**CRYO**	4 (9%)	7 (16%)	7 (16%)	4 (9%)	8 (18%)

All variables are presented as frequencies and percentages.
Abbreviations: ALD: alcohol-related liver disease, CRYO: cryptogenic/other, FU: follow-up, IALD: infectious/autoimmune liver disease, LTx: liver transplantation, MASH: metabolic dysfunction-associated steatohepatitis, MetALD metabolic and alcohol-associated liver disease, oHE: overt hepatic encephalopathy

Supplementary table 6. Variables adjusted for in Model B

	Multivariable linear regression analyses	Multivariable binary logistic regression analyses
**PHES**	Age<br>Years of school education<br>Inpatients<br>MELD<br>Sodium<br>Hemoglobin	Previous oHE episode<br>Age<br>Diabetes mellitus<br>Inpatients<br>MELD<br>Sodium<br>Hemoglobin
**CRT**	Previous oHE episode<br>Sex<br>Sodium<br>Albumin<br>Hemoglobin	Previous oHE-episode<br>Sex<br>Hemoglobin
**Stroop**	Age<br>Years of school education<br>CV disease<br>Sodium<br>Creatinine	Hemoglobin
**ANT**	Diabetes mellitus<br>German as native language<br>MELD<br>Hemoglobin	Age<br>Diabetes mellitus<br>German as native language
**ICT**	Age<br>Years of school education<br>CV disease	CV disease<br>TIPS
**CFF**	Child-Pugh-Score<br>Hemoglobin	Child-Pugh Score<br>Creatinine<br>Platelets

Abbreviations: ANT: Animal Naming Test, CFF: Critical Flicker Frequency, CRT: Continuous Reaction Time Test, CV cardiovascular, ICT: Inhibitory Control Test, MELD: model for end-stage liver, oHE: overt hepatic encephalopathy, PHES: Portosystemic Hepatic Encephalopathy Score, TIPS: transjugular intrahepatic portosystemic shunt

Supplementary figure 1. Box plots of mHE test results grouped by etiology

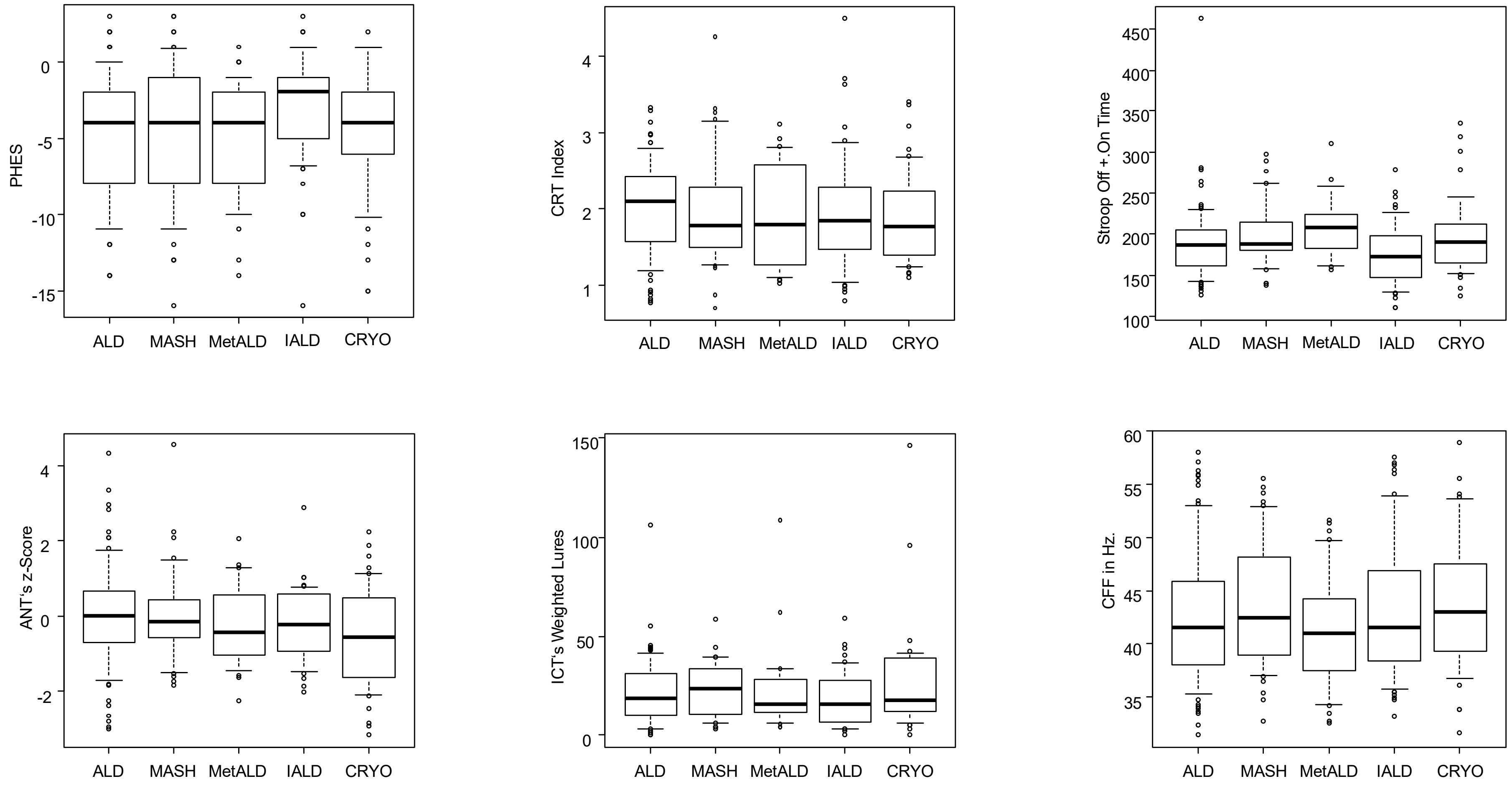

Abbreviations: ALD: alcohol-related liver disease, ANT: Animal Naming Test, CFF: Critical Flicker Frequency, CRT: Continuous Reaction Time Test, CRYO: cryptogenic/other, IALD: infectious/autoimmune liver disease, ICT: Inhibitory Control Test, MASH: metabolic dysfunction-associated steatohepatitis, MetALD metabolic and alcohol-associated liver disease, PHES: Portosystemic Hepatic Encephalopathy Score

Supplementary figure 2. Standardized log-rank statistics for the ideal cut-off value of PHES for predicting overt hepatic encephalopathy in 1 year

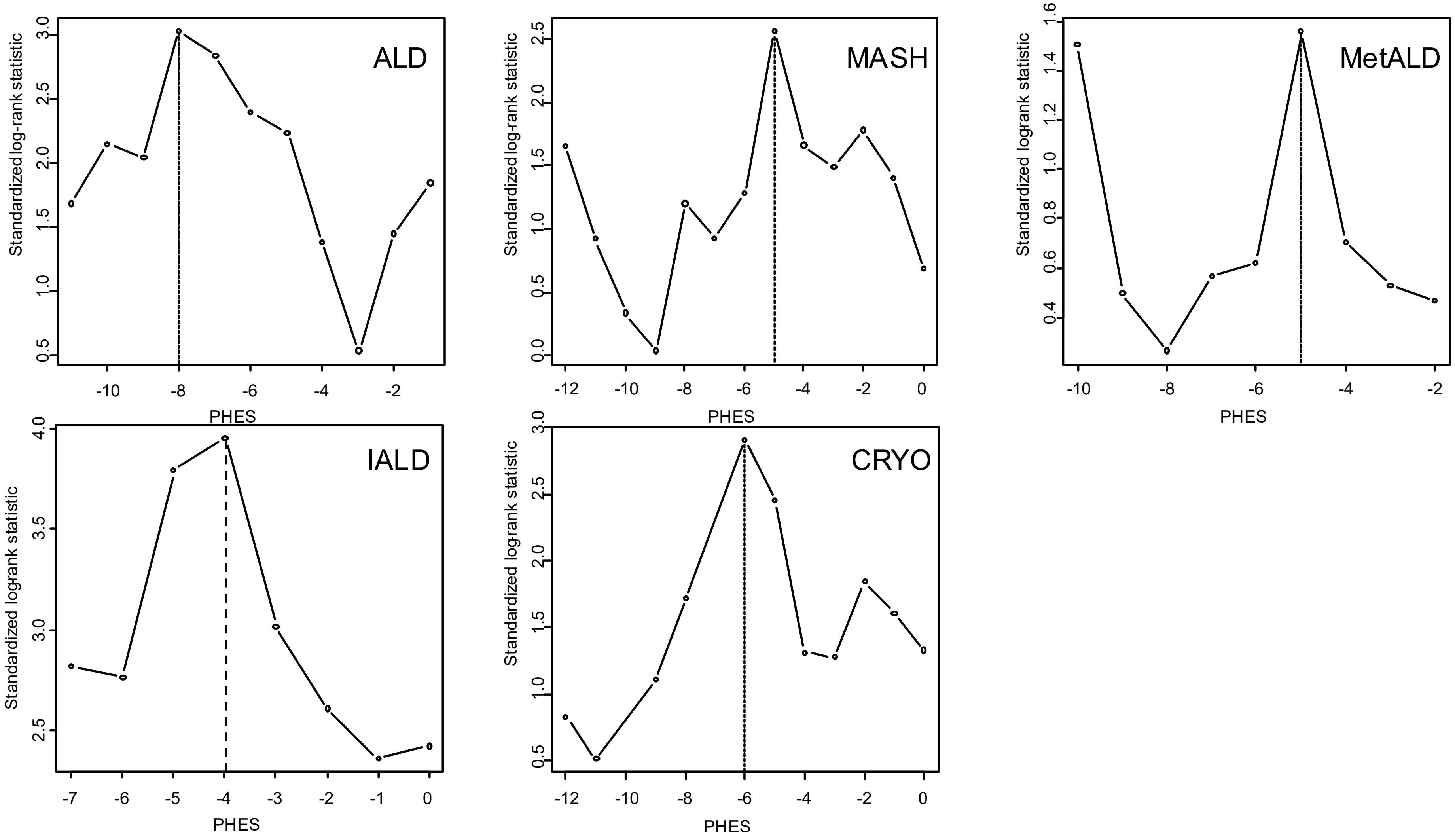

Abbreviations: ALD: alcohol-associated liver disease, CRYO: cryptogenic & others, IALD: infectious or autoimmune liver disease, MASH: metabolic dysfunction-associated steatohepatitis, MetALD: metabolic dysfunction and alcohol associated steatotic liver disease, HE: hepatic encephalopathy, PHES: Portosystemic Hepatic Encephalopathy Score

Supplementary figure 3. Directed acyclic graphs to visualize the associations between etiology and minimal hepatic encephalopathy test results

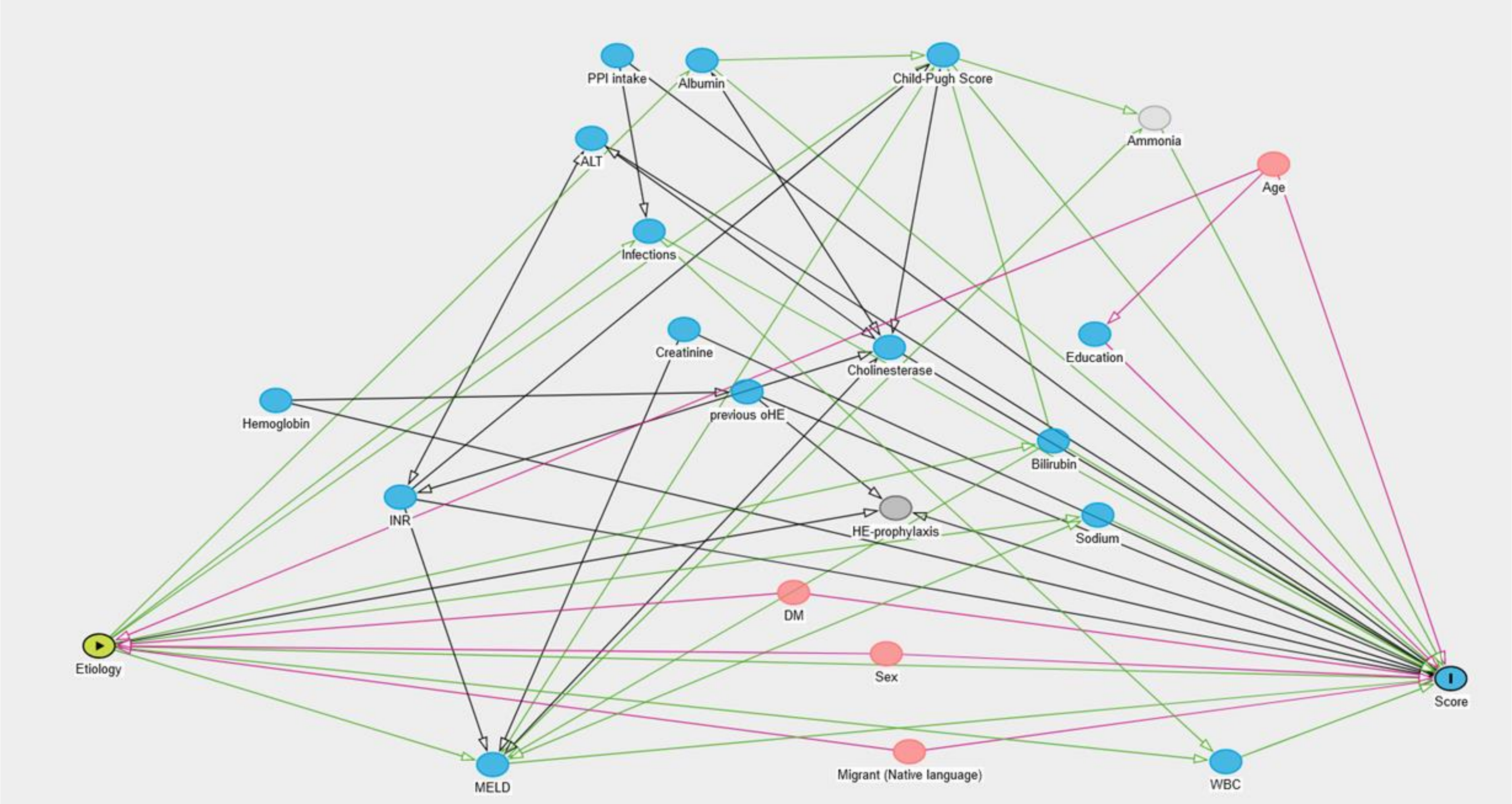

<u>Abbreviations:</u> ALT: Alanine aminotransferase, DM: diabetes mellitus, HE: hepatic encephalopathy, INR: international normalized ratio, MELD: model for end-stage liver disease oHE: overt hepatic encephalopathy, PPI: proton pump inhibitors, WBC: white blood cells
